# Rewired glycolysis by DTL accelerates oncometabolite L-lactate generation to promote breast cancer progression

**DOI:** 10.3389/fonc.2025.1583752

**Published:** 2025-05-05

**Authors:** Yuhao Liu, Jinting Li, Yiren Cao, Mengzhu Lv

**Affiliations:** ^1^ Key Laboratory of Carcinogenesis and Translational Research (Ministry of Education), Department of Radiation Oncology, Peking University Cancer Hospital and Institute, Beijing, China; ^2^ Key Laboratory of Carcinogenesis and Translational Research (Ministry of Education), Laboratory of Molecular Oncology, Peking University Cancer Hospital and Institute, Beijing, China

**Keywords:** DTL, glycolysis, L-lactate, breast cancer, progression

## Abstract

Breast cancer (BC) has become the leading cause of global cancer incidence. Despite therapeutic advances, a critical unmet need persists for identifying novel therapeutic targets. Our integrated bioinformatics analysis identified DTL, a component of the Cullin-RING ligase (CRL) E3 ubiquitin ligase family, as significantly upregulated in BC tissues. This upregulation correlated with poor patient prognosis, cancer stemness, and metabolic reprogramming, which was driven by genetic alterations such as gene amplification and reduced promoter methylation. Functional studies demonstrated that DTL promoted breast cancer cell proliferation and migration *in vitro* through glycolysis remodeling. Mechanistically, DTL positively regulated key glycolytic enzymes (HK2, ENO1, PKM2, and LDHA) independently of its canonical ubiquitin ligase activity and directly interacted with LDHA. Notably, exogenous L-lactate directly enhanced BC tumor growth and metastasis. Collectively, our findings reveal a non-canonical mechanism whereby DTL drives glycolysis to generate the oncometabolite L-lactate, which directly sustains breast cancer malignancy independent of protein degradation. The strong association between DTL upregulation and adverse clinical outcomes, coupled with its multifaceted regulatory roles in tumor biology, highlighting its therapeutic potential as a novel target in BC.

## Introduction

Breast cancer (BC) has now surpassed lung cancer to become the predominant cause of cancer incidence globally, with an estimated 2.3 million new cases, accounting for 11.7% of the total cancer caseload (1). Despite advances in multimodal therapies including surgical resection, chemotherapy, radiotherapy, endocrine therapy, and monoclonal antibody treatments that have improved patient outcomes, recurrence and mortality remain persistent challenges in BC management (2). This unmet clinical need underscores the urgency to identify novel therapeutic targets and decipher their molecular mechanisms to improve clinical outcomes.

DTL, Denticleless E3 ubiquitin protein ligase homolog, facilitates the assembly of the Cullin 4A-RING ubiquitin ligase (CRL4A) complex, comprising damage-specific DNA binding protein 1 (DDB1), DDB1 cullin4-associated factor (DCAF), the Cullin protein family member CUL4A, and the RING finger protein RBX1 (3). This complex catalyzes the direct transfer of ubiquitin from an E2 ubiquitin-conjugating enzyme to a lysine residue in the substrate, thereby initiating the ubiquitination process (4). Cullin-RING E3 ubiquitin ligases (CRLs) constitute the largest E3 ligase family and function as modular platforms that tightly regulate protein ubiquitination and degradation, which is essential for both physiological homeostasis and cancer progression (5). Human cells express seven cullin paralogs (CUL1/2/3/4A/4B/5/7), each forming distinct CRL complexes that selectively regulate specific protein substrates through spatiotemporal control (6). Emerging evidence reveals non-degradative ubiquitination roles of CRLs beyond their canonical proteolytic functions, expanding their regulatory scope in cellular physiology (7). Non-proteolytic ubiquitination now emerges as a critical regulator of fundamental processes including signal transduction, genomic stability maintenance, and cell cycle control (8). Moreover, dysregulation of these non-canonical ubiquitination pathways has been implicated in oncogenic transformation (7).

Metabolic reprogramming, a well-established cancer hallmark, manifests in BC through enhanced glycolysis and anabolic flux to sustain uncontrolled proliferation and survival (9, 10). This metabolic shift not only fuels the energy and biosynthetic demands of cancer cells but also enhances their resistance to oxidative stress and therapy-induced cell death (11). Recent studies implicate CRLs, particularly DTL-containing complexes, in orchestrating cancer metabolism through the regulation of glycolytic enzyme networks (12–14). Deciphering E3 ligase-metabolic pathway crosstalk in BC could uncover actionable targets for precision oncology.

In our study, bioinformatics analysis identified DTL as the most upregulated CRL in BC, with its expression strongly correlating with clinical survival outcomes. Further characterization revealed DTL-associated genetic alterations, and metabolic reprogramming. Experimental validation confirmed DTL upregulation in BC and its prognostic relevance. Mechanistically, DTL is essential for maintaining the expression of glycolytic enzymes. Overexpression of DTL drives L-lactate accumulation to promote tumor progression, nominating DTL as a dual diagnostic and therapeutic target in BC.

## Materials and methods

### Screening for DTL in breast cancer

Bioinformatic screening was performed using UbiNet 2.0 (15) and GEPIA2 (16) to identify CRLs with significant upregulation in BC. UbiNet 2.0 provides a comprehensive database of E3-substrate interactions and tools for analyzing the ubiquitination network. GEPIA2 is an analytical platform for RNA sequencing expression data from 9,736 tumor and 8,587 normal samples, sourced from The Cancer Genome Atlas (TCGA) and Genotype-Tissue Expression (GTEx) projects.

### DTL expression analysis

Pan-cancer DTL expression profiling was conducted using UALCAN (17) with the TCGA pan-cancer cohort (24 tumor types). The TCGA-BRCA cohort (n=1,085) was subsequently analyzed for disease-specific expression patterns. Comparative analysis with normal controls was performed using 291 non-tumor samples (TCGA, n=112; GTEx, n=179) for mRNA expression. Proteomic validation was executed through the Clinical Proteomic Tumor Analysis Consortium (CPTAC) database with data visualization implemented.

### Association of DTL with clinical characteristics

BC cases from the TCGA cohort were stratified according to clinicopathological parameters including cancer stages, age, molecular subclasses, and nodal metastasis status. Intergroup differential expression analysis of DTL was performed using non-parametric Kruskal-Wallis test with Benjamini-Hochberg correction (FDR<0.05).

### Survival analysis

Survival analysis was performed using the breast cancer module in Kaplan-Meier Plotter (18), which integrates multiple cohorts of BC patients. Associations between DTL expression and survival endpoints including overall survival (OS), relapse-free survival (RFS), and distant metastasis-free survival (DMFS) were evaluated. Patients were dichotomized into high/low DTL groups using median expression thresholds. Cox proportional hazards regression was applied to calculate hazard ratios (HRs) with 95% confidence intervals, and statistical significance was assessed via log-rank test (two-tailed, *α*=0.05).

### Gene alteration analysis

Statistical analysis of DTL genetic alterations was performed using cBioPortal (19) across pan-cancer cohorts. The 3D protein structure and mutation hotspots of DTL were also analyzed using cBioPortal. Tumor mutational burden (TMB) and mutant allele tumor heterogeneity (MATH) across pan-cancer cohorts were calculated using TCGA MAF files analyzed in R/maftools (20). TMB was defined as non-synonymous mutations per coding megabase, while MATH quantified intra-tumor heterogeneity via median absolute deviation of allele frequencies. Pan-cancer validation leveraged published TCGA benchmarks, with results visualized using ggplot2 package in R environment for cross-cancer comparisons. Promoter methylation levels of DTL in BC were assessed via UALCAN. The correlation between DTL expression and copy number variation (CNV) was investigated using GSCA (21).

### Analysis of DTL’s role in cellular signaling and stemness

While exploratory gene co-expression analyses were performed using TIMER2.0 (22), correlations with |Rho| < 0.3 were excluded from final interpretations to avoid overstatement of biological relationships. Proteomic data from the CPTAC database were analyzed via UALCAN to evaluate associations between DTL expression and cellular signaling pathways/regulatory molecules.

### Exploring the impact of DTL on metabolic reprogramming

Metabolic pathway analysis was conducted via TIMER2.0 to assess DTL expression correlations with metabolic reprogramming-related genes. The TCGA-BRCA cohort was dichotomized into DTL high/low groups by median expression, with differential expression of key metabolic enzymes analyzed using Wilcoxon rank-sum tests. TP53 mutation status associations with DTL expression and metabolic enzymes were evaluated in BC using TIMER2.0, stratified by mutation status (wild-type versus mutated).

### Cell culture

The human breast cancer (BC) cell lines MDA-MB-231 and CAL-51 were obtained from American Type Culture Collection (Manassas, USA) and preserved in our laboratory. These BC cells were cultured in DMEM/RPMI 1640 culture medium (Lonza, Switzerland) supplemented with 10% FBS (Gibco, USA), 100 units/mL penicillin, and 100 mg/mL streptomycin (Invitrogen, USA) in an incubator with 5% CO_2_ at 37°C. All these BC cells have been identified using short tandem repeat (STR) profiling.

### Clinical specimens

The BC specimens were acquired from Shanghai Outdo Biotech Co. Ltd., and informed consent was obtained from all subjects and/or their legal guardians. All methods were carried out in accordance with relevant guidelines and regulations, and all experimental protocols were approved by the Research Ethics Board of Peking University Cancer Hospital.

### siRNAs transfection

genOFF h-DTL_1999A was provided by RiboBio (SIGS0003394-1, China) and used to siRNAs transfection against *DTL* according to the manufacturer’s instructions. Lipofectamine™ 2000 Transfection Reagent was purchased from Invitrogen (11668019, USA). The siRNAs used in this study were listed in **Supplementary Table S1**.

### Immunohistochemical staining

The tissue specimens containing tumor tissues (n=160) and adjacent tissues (n=100) of primary BC (HBre-Duc170Sur-01, HBre-Duc090Sur-01) were provided by Shanghai Outdo Biotech Co. Ltd., as well as their corresponding pathological information. Then the tissue chips were deparaffinized and hydrated, followed by antigen retrieval through boiling for 3 minutes in Tris-EDTA buffer (pH 9.0). The tissues were blocked using 10% normal goat serum (ZSGB-BIO, China) and subsequently incubated with anti-DTL antibody (1:100, Abcam, ab72264) at 4°C overnight. After that, incubated the sections with the secondary antibody and stained using DAB (Dako, Denmark). The expression of DTL protein was evaluated according to the staining intensity as following: 0, negative; 1, weak; 2, strong; 3, very strong. For staining area, scored as following pattern: 0, <5%; 1, 5–25%; 2, 26–50%; 3, 51–75%; 4, >75%. The product of staining intensity score and staining area score is defined as “H score”. H scores of 0–4 were defined as “low expression” and scores of 6–12 as “high expression”.

### CCK-8 assay

Cell viability was measured using CCK-8 kit (RM02823, ABclonal, China). In brief, 3×10^3^ cells were counted and seeded on 96-well culture plates. At 0, 24, 48, and 72 hour, added 100 μL 10% CCK-8 solution and incubated for 1–4 hours protecting from light. Recorded the OD values at 450 nm using a microplate reader (Tecan, Austria).

### Clone formation assay

2×10^3^ BC cells were counted and plated on 6-well culture plates. Incubated these cells with complete culture medium for about two weeks and then fixed the colonies with 100% methanol solution for 10 minutes at room temperature. Stained these colonies using 0.1% crystal violet solution for 20 minutes and visualized the grown colonies by a stereomicroscope (Leica, Germany).

### Cell migration assay

To measure the cell migration, about 2×10^5^ BC cells were counted, then resuspended with 200 μL culture medium without FBS and seeded into the top chamber of a 24-well insert (3422, Corning, USA). Added 1 mL culture medium containing 20% FBS to the bottom chamber and changed into 100% methanol solution within 24 hours to fix migrated cells for 10 minutes. Stained these migrated cells with 0.1% crystal violet solution for 20 minutes. Taked photographs and analyzed these migrated cells using a stereomicroscope (Leica, Germany).

### Western blot

Proteins were extracted using RIPA buffer (P0013E, Beyotime, China) supplemented with protease and phosphatase inhibitors (Roche, Germany). BCA Protein Assay Kit (P0011, Beyotime, China) was used to quantify the extracted protein. 20-30 μg protein was separated using 10% SDS-PAGE gels (P2012, New Cell & Molecular Biotech, China) and transferred to PVDF membranes (Millipore, USA). All the primary antibodies were listed in **Supplementary Table S2**. Protein bands were detected using the Amersham Imager 600 (GE Healthcare, USA).

### Glucose uptake assay

2-NBDG (HY-116215, MedChemExpress, USA) was used to detect relative glucose uptake of BC cells. In brief, about 1×10^6^ BC cells were seeded on 6-well culture plates and then changed into culture medium without glucose for 16–24 hours. Added 100 μM 2-NBDG solution to these cells and incubated them for 30 minutes at 37°C. Washed the cells with 1×PBS twice and then analyzed the fluorescence intensity using BD Accuri™ C6 flow cytometer (BD Biosciences, USA).

### L-lactate assay

Intracellular L-lactate production was measured using L-Lactate Assay Kit (K627, Biovision, USA) according to the manufacturer’s instructions.

### Seahorse assay

The measurement of extracellular acidification rate (ECAR) in BC cells was performed using Seahorse XF Glycolysis Stress Test Kit (103020, Agilent Technologies, USA) and analyzed using the XFe96 analyzer (Agilent Technologies, USA) according to the manufacturer’s instructions.

### Cell cycle assay

For cell cycle analysis, 1×10^6^ cells were fixed with 70% ethanol at 4°C overnight and then washed the cells with 1×PBS twice. Stained the cells with PI (550825, BD Biosciences, USA) for 20 minutes at room temperature and subsequently analyzed their cell cycle distribution or DNA content using Accuri™ C6 flow cytometer (BD Bioscience, USA).

### Proximity Ligation Assay

PLA assays were performed using the *in-situ* Proximity Ligation Assay kit (Sigma-Aldrich, DUO92008) following the manufacturer’s protocol and our previously described method (23). Briefly, approximately 2 × 10^5^ MDA-MB-231 and CAL-51 cells were seeded in confocal dishes. After 24 hours, cells were washed with 1× PBS and fixed with ice-cold methanol for 15 min. Following three PBS washes, 50 μL of Blocking Solution was added, and cells were incubated for 1 h at 37°C. After blocking, primary antibodies of anti-DTL (Proteintech; 12896-1-AP; 1:50) paired with either anti-ENO1 (Proteintech; 67187-1-Ig; 1:50) or anti-LDHA (Proteintech; 66287-1-Ig; 1:50) were diluted in Antibody Diluent, and 50 μL of the antibody solution was added to the cells for overnight incubation at 4°C. Subsequently, cells were washed twice with 1× Wash Buffer A, followed by incubation with 50 μL of diluted PLUS and MINUS PLA probes for 1 h at 37°C. After another wash with 1× Wash Buffer A, ligation was performed by incubating cells with 50 μL of ligation solution (Ligase in 1× Ligation Buffer, 1:40 dilution) for 30 min at 37°C. For amplification, cells were washed twice with 1× Wash Buffer A and incubated with 50 μL of polymerase solution (Polymerase in 1× Amplification Buffer, 1:80 dilution) for 100 min at 37°C. Next, cells were washed twice with 1× Wash Buffer B and once with 0.01× Wash Buffer B. Finally, PLA Mounting Medium with DAPI was applied, and after a 15-min incubation, images were acquired using a confocal microscope (Leica ST2).

### Statistical analysis

Bivariate correlations between continuous variables were evaluated using Spearman’s rank correlation coefficient. Categorical variables were compared between groups using *χ* ^2^ tests or Fisher’s exact tests (applied when expected cell counts <5). Non-normally distributed continuous variables were analyzed via Mann-Whitney U test (two-group medians comparison). Kaplan-Meier survival curves with log-rank testing were generated to assess DTL expression-survival associations. All statistical analyses and data visualizations were performed using R statistical computing environment (v4.3.1). Two-tailed tests were applied throughout, with statistical significance defined as p<0.05 (denoted by asterisks in figures).

## Results

### DTL expression patterns and prognostic significance in breast cancer

**Figure 1:**
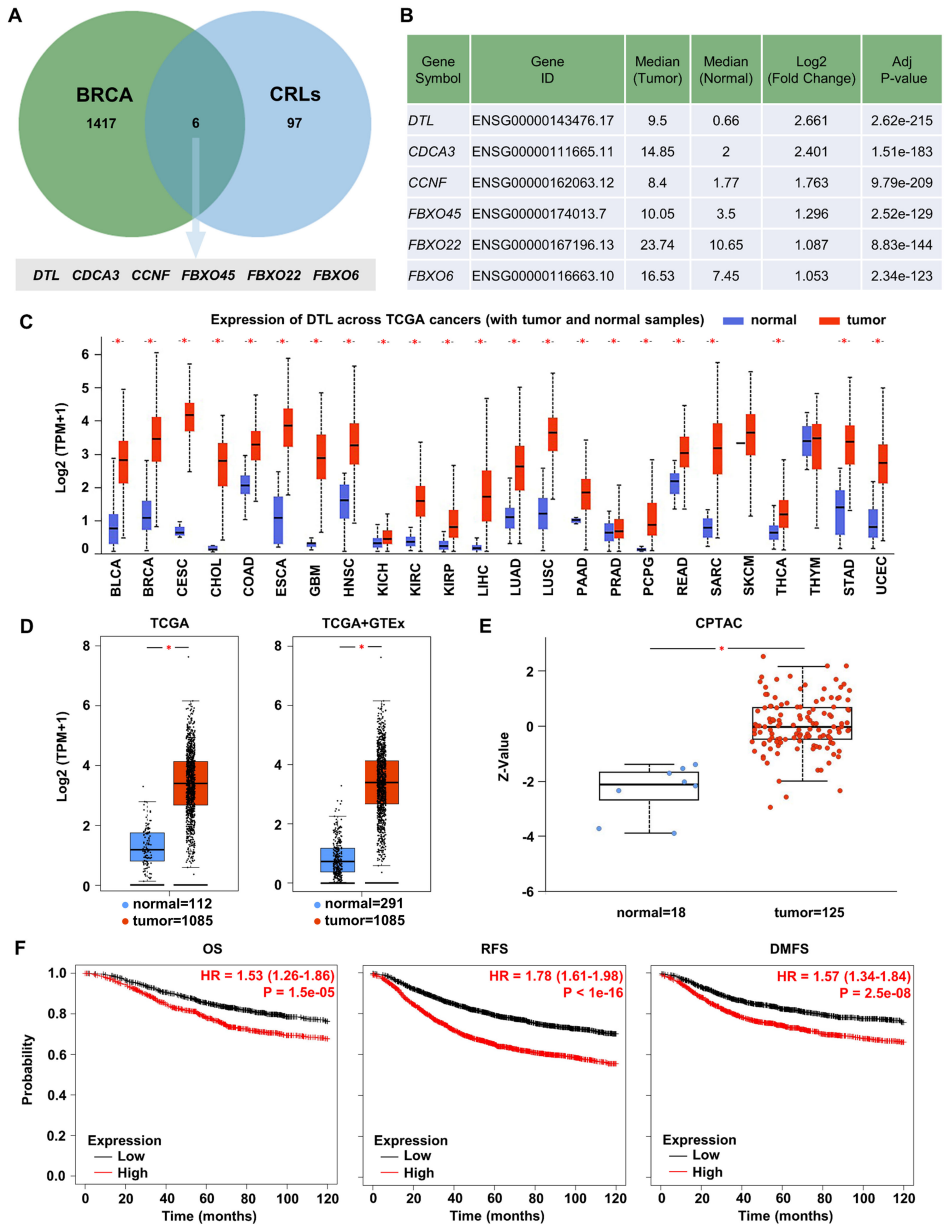
DTL is highly expressed in breast cancer and significantly associated with the prognosis of breast cancer. **(A)** Venn diagram identifying CRLs upregulated in BRCA. Green circle: 1423 genes upregulated in BRCA. Blue circle: 103 CRLs from reference databases. Intersection: 6 CRLs significantly upregulated in BRCA and their gene details have been listed in **(B)**. **(C)** Box plot showing the expression of DTL mRNA in various TCGA cancer samples including breast cancer. **(D)** Analysis for TCGA and GTEx database determining the expression of DTL mRNA in breast cancer samples and normal tissue samples. **(E)** Box plot demonstrating the expression of DTL protein in breast cancer tissues and normal breast tissues. **(F)** Kaplan-Meier curves analysis suggesting the correlation of the survival outcome including overall survival (OS), relapse-free survival (RFS), and distant metastasis-free survival (DMFS) of breast cancer patients with DTL expression.

Integrated analysis of GEPIA2 differential expression data and UbiNet 2.0 ubiquitination networks identified DTL among six CRLs showing maximal BC/normal expression differentials (**Figures 1A, B**). Transcriptomic and proteomic validation confirmed tumor-specific DTL upregulation across BC specimens (**Figures 1C–E**). Clinicopathological stratification demonstrated heterogeneous DTL expression patterns across various clinical parameters, including ethnicity, TP53 mutation status, cancer stage, and age. Notably elevated DTL expression was observed in Asian patients and TP53-mutant cases, whereas stage progression (stage 1 to stage 2) correlated with reduced expression. Younger age cohorts showed comparatively higher levels (**Supplementary Figure S1**). Survival analysis of BC patients revealed significant prognostic associations between elevated DTL expression and adverse clinical outcomes. The results indicated that increased DTL expression was significantly associated with poor OS, RFS, and DMFS outcomes, highlighting its potential as a strong prognostic indicator in BC (**Figure 1F**). Furthermore, we investigated the impact of DTL expression on OS (**Supplementary Figure S2**), RFS (**Supplementary Figure S3**), and DMFS (**Supplementary Figure S4**) in BC patients with distinct clinical characteristics. The results demonstrated that elevated DTL expression was associated with poorer prognosis in ER-positive or HER2-negative patients. In contrast, no significant association between DTL expression levels and survival outcomes was observed in patients with basal subtype or grade 3 tumors.

### Genomic and epigenetic analysis of DTL in breast cancer

**Figure 2:**
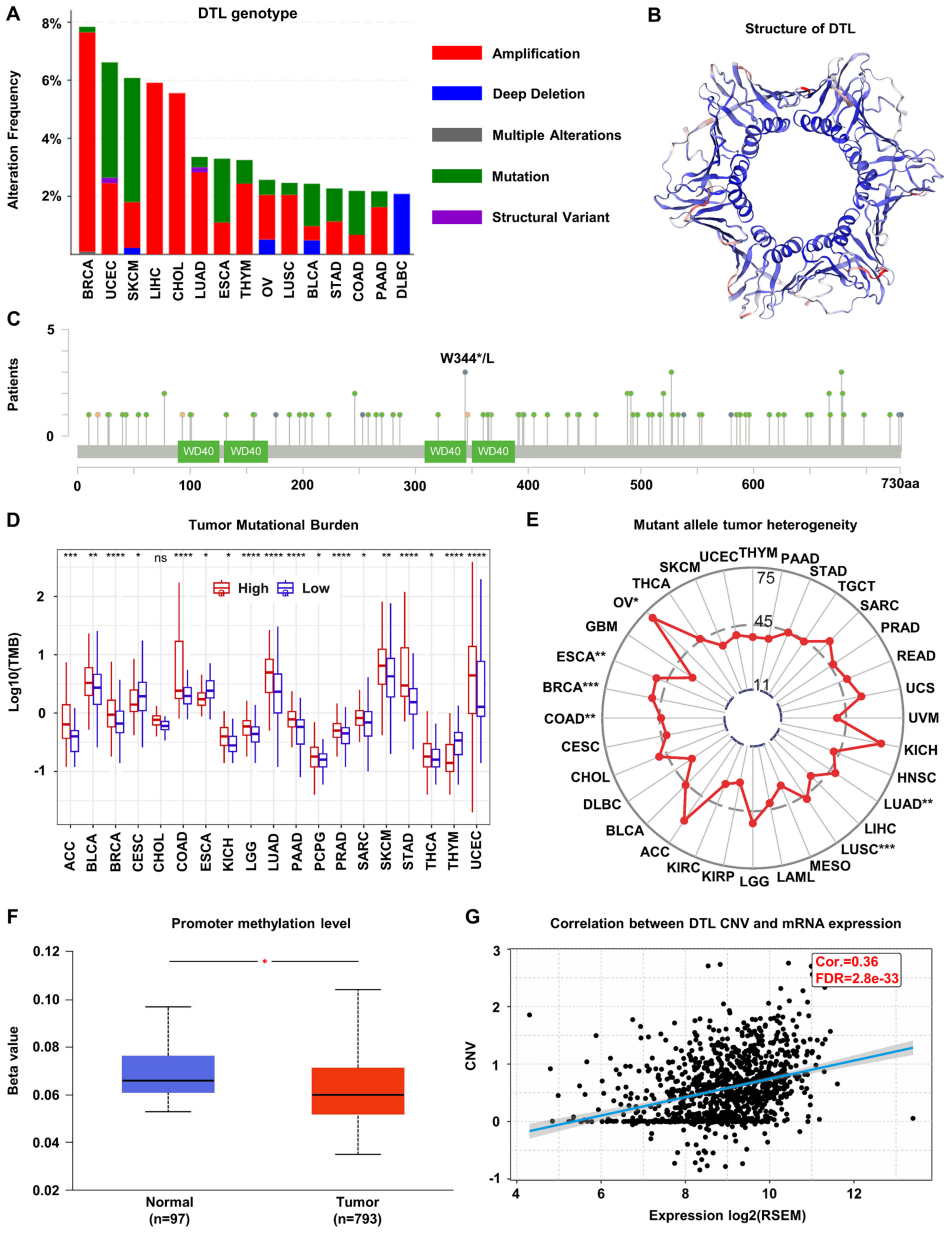
Genomic alterations of DTL in breast cancer. **(A)** The alternations of DTL genotype in breast cancer. **(B)** Schematic diagram of the three-dimensional structure of DTL protein. **(C)** Common mutation sites in the DTL gene including W344*/L. **(D)** Tumor mutational burden in various cancers including breast cancer with high and low DTL expression groups. **(E)** Mean MATH scores of mutation allele tumor heterogeneity for high and low DTL expression groups in various cancers including breast cancer. **(F)** Box plot indicating the promoter methylation level of DTL gene in breast cancer tissues and normal tissues. **(G)** Scatter plot showing the correlation between DTL expression and its copy number variation in breast cancer.

Genomic profiling of BC specimens identified frequent genetic alterations in the DTL locus, with amplification representing the predominant alteration type (**Figure 2A**), suggesting a potential driver for DTL overexpression in BC. To further understand the structural implications of DTL mutations, we characterized the three-dimensional protein structure of DTL and identified a common mutation site, W334*/L (**Figures 2B, C**). This residue localized to a conserved functional domain, suggesting possible implications for protein integrity and tumor pathogenesis. DTL expression showed positive correlations with TMB and MATH scores (**Figures 2D, E**), which may reduce genomic stability and increase mutational susceptibility. Primary tumors showed lower methylation levels at the DTL promoter compared to normal adjacent tissues (**Figure 2F**). Additionally, DTL CNV demonstrated a statistically significant, albeit moderate, positive correlation with transcriptional output (Cor. = 0.36, FDR=2.8e-33) (**Figure 2G**).

### DTL’s multifaceted impact on breast cancer biology

**Figure 3:**
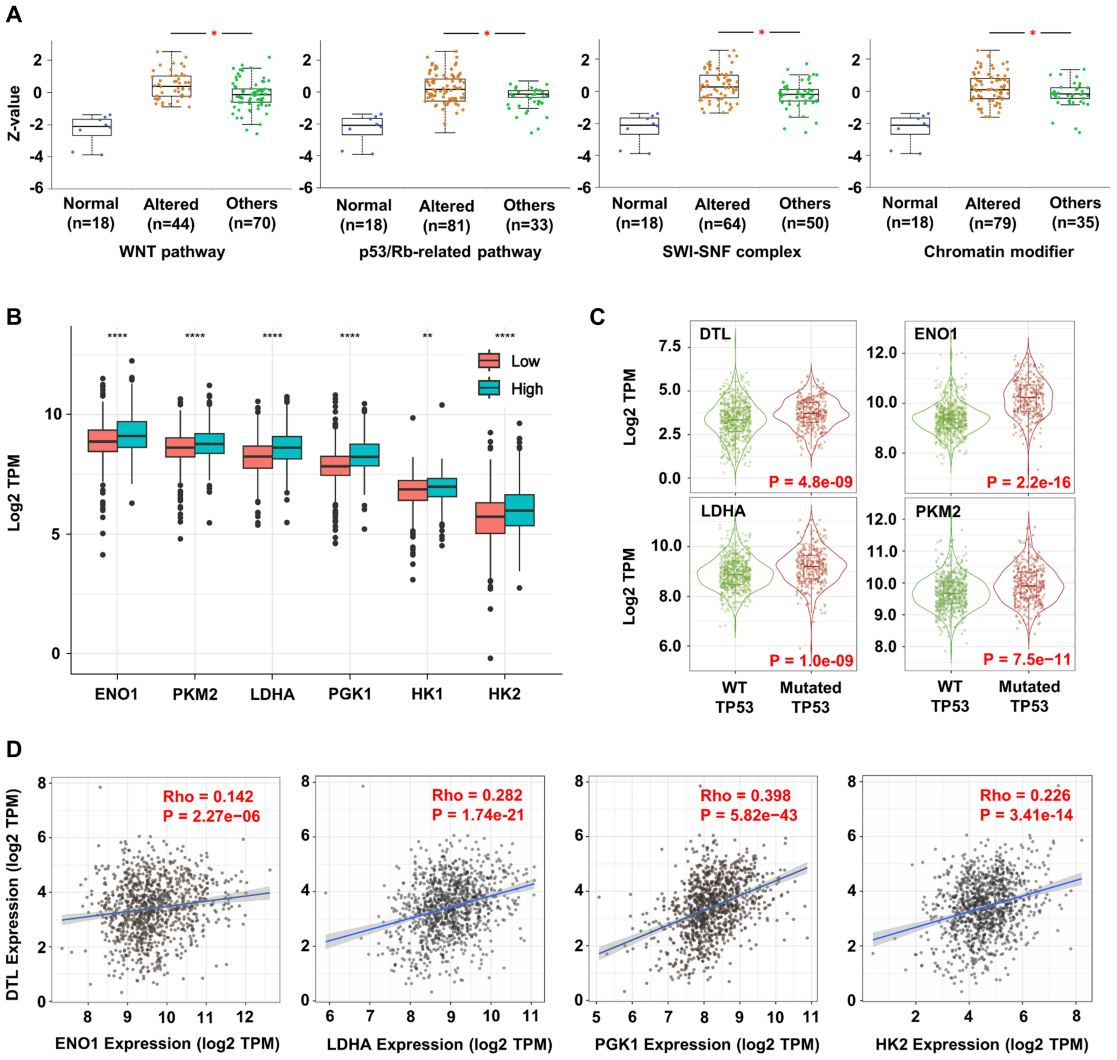
DTL promotes oncogenesis and progression of breast cancer by reprogramming metabolism and enhancing stemness of tumor cells. **(A)** Box plots showing the DTL expression in breast cancer samples with alternative stemness-related signaling and normal pathways. **(B)** The expression of key enzyme genes in glycolytic pathway (such as ENO1, PKM2, LDHA, PGK1, HK1, HK2) in breast cancer tissues with low and high DTL expression. **(C)** The violin plots indicating the expression of DTL, ENO1, LDHA, and PKM2 in breast cancer samples with mutant TP53 compared to samples with wild-type TP53. **(D)** Scatter plots demonstrating the correlation between the expression of DTL and multiple key enzymes in glycolytic pathway (such as ENO1, LDHA, PGK1, HK2).

Significant correlations were observed between proteomic expression of DTL and alterations in cancer-related signaling pathways, such as the WNT and p53/Rb pathways (**Figure 3A**). These pathways regulate core oncogenic processes, consistent with DTL’s association with BC progression. Extended pathway interaction analysis was also provided (**Supplementary Figure S5**). Further analysis highlighted the metabolic effects of DTL on BC cells. Elevated DTL expression correlated with the upregulation of glycolytic enzyme including ENO1, PKM2, LDHA, PGK1, HK1, and HK2 (**Figure 3B**). The positive correlation between DTL expression and these genes suggests that DTL may enhance aerobic glycolysis in BC cells, a metabolic shift often associated with cancer progression. TP53-mutant BC exhibited coordinated overexpression of DTL and glycolytic enzymes versus wild-type (**Figure 3C**). The TP53-DTL-glycolysis triad may reflect established genomic-metabolic crosstalk in BC biology. Further analysis confirmed cohesive co-expression between DTL and glycolytic nodes (**Figure 3D**). The correlation landscape of DTL expression with other glycolytic enzymes has also been investigated (**Supplementary Figure S6**).

### The clinical significance of elevated DTL expression in breast cancer

**Figure 4:**
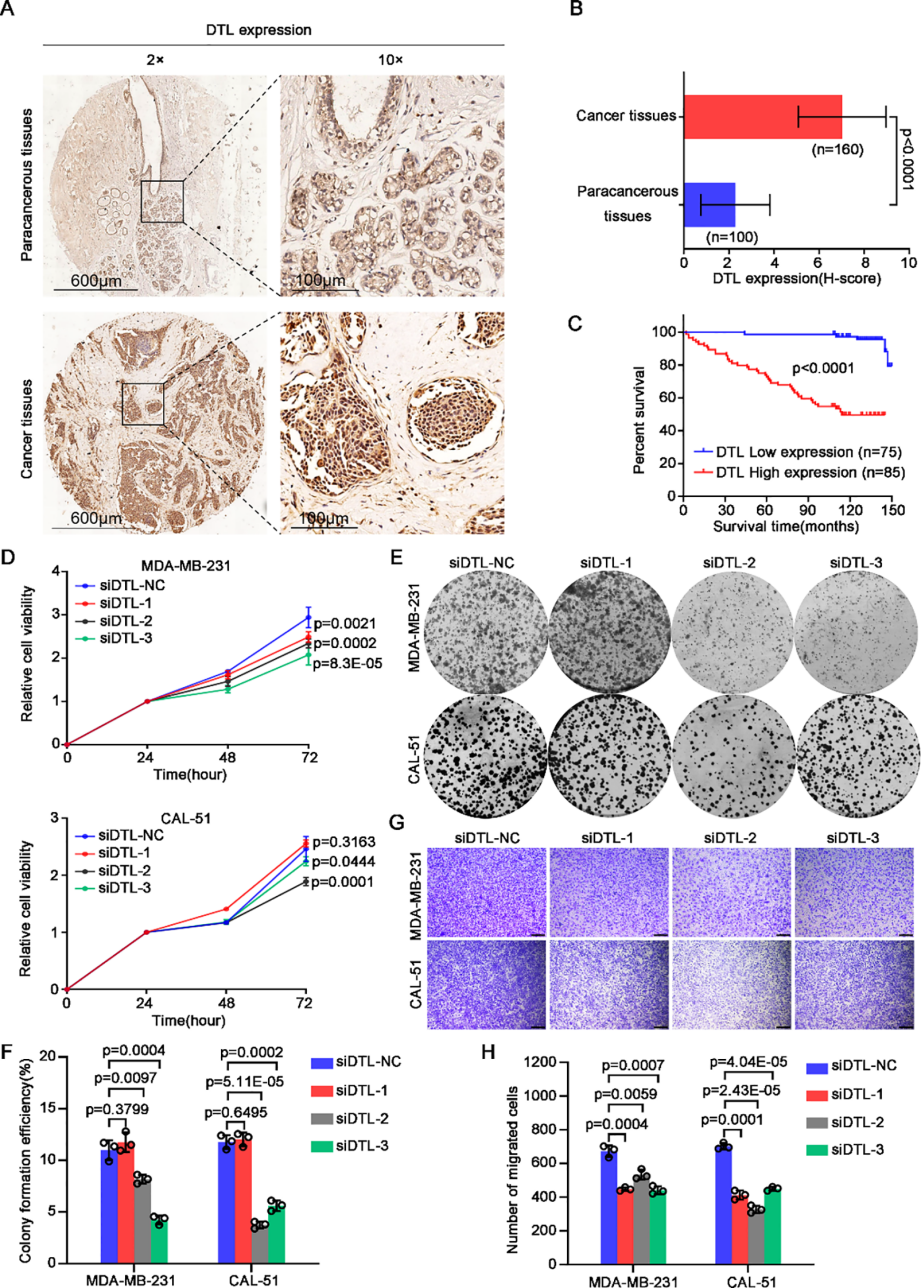
DTL drives the malignant development of breast cancer. **(A)** Immumohistochemical staining analysis showing the expression of DTL in 100 paracancerous tissues and 160 tumor tissues of breast cancer patients and the H-scores were graphed as a histogram in **(B)**. **(C)** Kaplan-Meier curves analysis indicating the correlation between breast cancer patients’ survival time and DTL protein expression. **(D)** CCK-8 assay measuring the relative cell viability of MDA-MB-231 and CAL-51 cells when DTL was knocked down using its siRNAs. **(E)** Representative micrographs of the grown colonies formed by MDA-MB-231 and CAL-51 cells after DTL depletion and the histogram in **(F)** showing their corresponding colonies-forming efficiencies. **(G)** Transwell assay indicating changed migrated capacity of DTL knocking-down MDA-MB-231 and CAL-51 cells and the histogram in **(H)** displaying the number of migrated cells. Scale bar, 100 μm. Data in **B**, **D**, **F** and **H** were presented as mean ± S.D and two-tailed Student’s *t*-test was used for statistical analysis.

To preliminarily assess DTL protein distribution patterns in BC tissues, we performed semi-quantitative immunohistochemistry (IHC) analysis on 160 tumor specimens and 100 matched paracancerous controls. While IHC provides spatial resolution rather than absolute quantification, the observed relative staining intensity (**Figures 4A, B**) suggests increased DTL expression in tumor versus normal epithelium. We subsequently investigated the impact of abnormal DTL expression on BC patients’ prognosis. We performed survival analysis according to the pathological information from these BC patients and Kaplan-Meier curves demonstrated that higher DTL protein level was significantly associated with shorter survival time of these BC patients (**Figure 4C**). In addition, we explored the correlation of DTL expression with clinicopathologic features in 160 primary BC cases. Surprisingly, high DTL protein level was associated with a poor 5-year survival rate, an advanced clinical stage and pregestational hormone (HR) status (**Table 1**). Hence, dysregulated DTL expression was closely related to poor prognosis of these BC patients.

**Table 1:** Association of DTL expression with clinicopathologic features in 160 primary breast cancer cases.

Features	Total	DTL expression		P value
		Low frequency	High frequency	
Survival				5e-05
≤5 years	24	1(4.2%)	23(95.8%)	
>5 years	136	74(54.4%)	62(45.6%)	
Tumor stage				0.037
Early stage 1-2	45	27(60.0%)	18(40.0%)	
Advanced stage 3-4	115	48(41.7%)	67(58.3%)	
Lymph-node metastasis				0.056
Yes	94	50(53.2%)	44(46.8%)	
No	66	25(37.9%)	41(62.1%)	
ER				0.139
Positive	108	55(50.9%)	53(49.1%)	
Negative	52	20(38.5%)	32(61.5%)	
PR				0.037
Positive	95	51(53.7%)	44(46.3%)	
Negative	65	24(36.9%)	41(63.1%)	
HER2				0.069
Positive	52	19(36.5%)	33(63.5%)	
Negative	108	56(51.9%)	52(48.1%)	

### DTL is essential to cell proliferation and migration of breast cancer

To investigate the regulatory effect of DTL during BC progression, we depleted the expression of DTL in MDA-MB-231 and CAL-51 cells using siRNAs. Notably, both cell viability and colony formation ability of these breast cells were downregulated dramatically after DTL knockdown (**Figures 4D–F**). Additionally, decreased DTL expression also contributed to reduced migrated potential of BC cells (**Figures 4G, H**). Given data indicated that DTL indeed plays an important role in the regulation of BC progression.

### DTL rewires glycolysis of breast cancer cells

**Figure 5:**
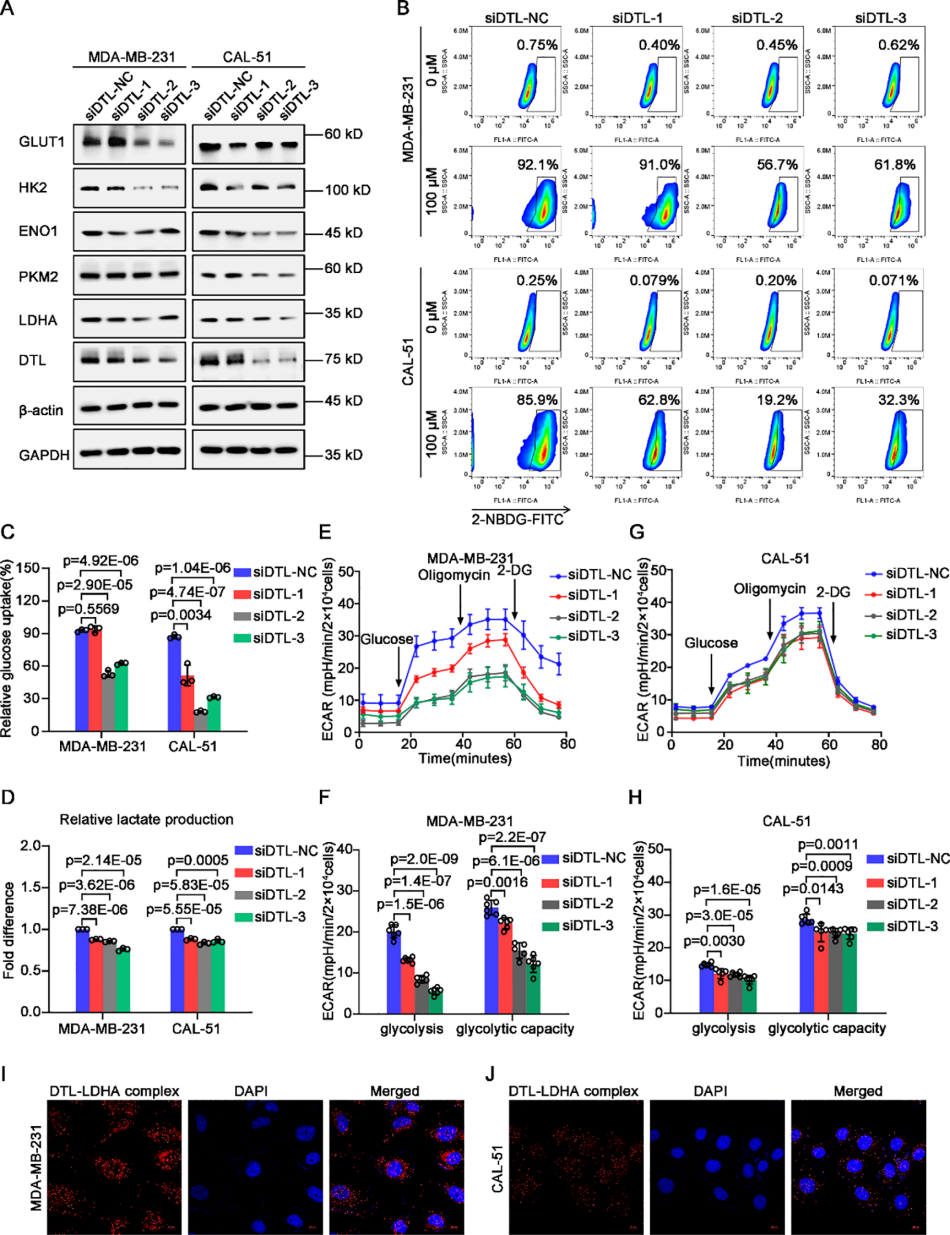
DTL is responsible for reprogrammed glycolysis of breast cancer cells. **(A)** Western blot analysis demonstrating the expression of glycolysis-associated transporter and enzymes in DTL-depleted and their control MDA-MB-231 and CAL-51 cells. GAPDH and β-actin were used as internal references. **(B)** Flow cytometry analysis for glucose uptake in DTL-knocked down and their control MDA-MB-231 and CAL-51 cells using 100 μM 2-NBDG. **(C)** Histogram showing alternative ability of glucose uptake after DTL depletion. **(D)** Histogram indicating intracellular L-lactate content of DTL-depleted and control MDA-MB-231 and CAL-51 cells. **(E)** ECAR curves exhibiting changed glycolysis of MDA-MB-231 cells when DTL was knocked down. **(F)** Histogram illustrating their alternative glycolysis and glycolytic capacity. **(G)** ECAR curves of DTL-knocked down and control CAL-51 cells and their glycolysis and glycolytic capacity was graphed as a histogram in **(H)**. **(I, J)** Proximity ligation assay (PLA) indicating the direct interaction of DTL and LDHA in MDA-MB-231 and CAL-51 cells. Nuclei were stained with DAPI. Scale bar, 10 μm. Data in **(C**-**H)** were the mean ± S.D of three independent experiments. Two-tailed Student’s *t*-test.

Based on the significant correlation between DTL status and glycolytic enzymes in our previous data, we further verified the role of DTL on glycolysis (**Supplementary Figure S7A**). Western blot analysis showed that the expression of glycolytic enzymes including HK2, ENO1, PKM2 and LDHA were down-regulated remarkably after DTL depletion in MDA-MB-231 and CAL-51 cells, as well as GLUT1, a transporter of glucose (**Figure 5A**). Glucose uptake assay also confirmed that intracellular glucose contents were decreased obviously cause of DTL knockdown using 2-NBDG staining, a glucose analogue labeled with FITC (**Figures 5B, C**). Furthermore, depleted DTL led to depressed intracellular L-lactate levels of these BC cells (**Figure 5D**). Also, as the seahorse data shown, the extracellular acidification rate (ECAR) curve was down-regulated dramatically when DTL was knocked down in both MDA-MB-231 and CAL-51 cells, involved in reduced glycolysis and glycolytic capacity (**Figures 5E–H**). Additionally, as the proximity ligation assay (PLA) data shown, DTL could interact directly with some glycolytic enzymes such as ENO1 and LDHA, especially for LDHA, with strong binding potential for DTL (**Figures 5I, J**; **Supplementary Figure S7B**). Collectively, all above-mentioned data demonstrate that DTL is a key determinant of reprogrammed glycolysis of BC cells.

### Alternative L-lactate production driven by DTL is responsible for proliferation and migration of breast cancer cells

**Figure 6:**
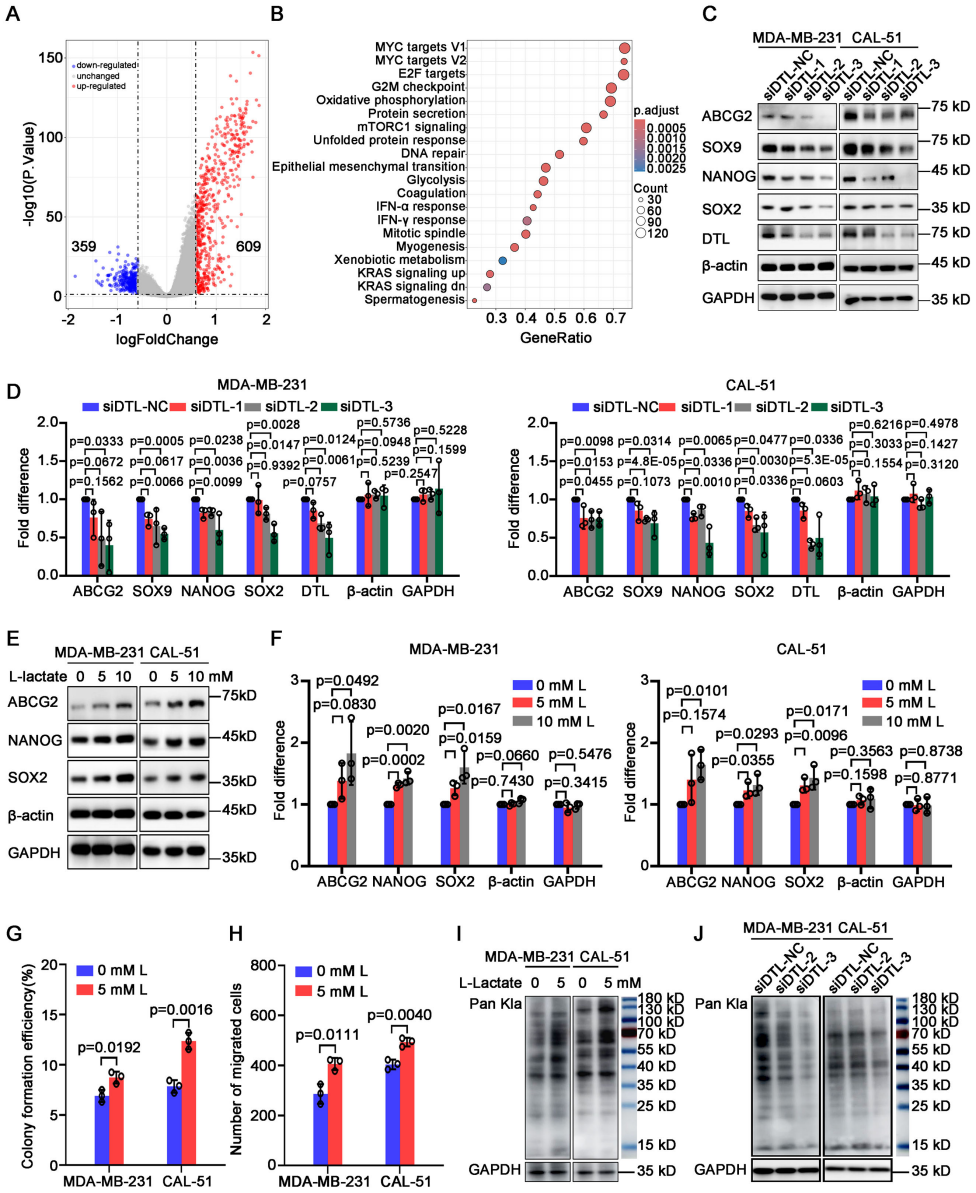
L-lactate supports cell proliferation and migration of breast cancer depending on DTL. **(A)** Volcano plot showing differentially expressed genes in breast cancer patients with low and high DTL expression. **(B)** Dot plot indicating the alternative signal pathways of KEGG enrichment analysis. **(C)** Western blot analysis for the expression of stemness-associated genes in MDA-MB-231 and CAL-51 cells after DTL knockdown. **(D)** Histogram showing the expression of indicated molecules after DTL knockdown in MDA-MB-231 and CAL-51 cells. **(E)** Immunoblotting analysis for the expression of stemness-related indicators in MDA-MB-231 and CAL-51 cells treated with different concentrations of L-lactate. **(F)** Histogram showing the levels of indicated molecules in MDA-MB-231 and CAL-51 cells treated with 5 mM L-lactate. **(G)** Histogram indicating the colony-forming efficiency of MDA-MB-231 and CAL-51 cells with 5 mM L-lactate treatment. **(H)** Histogram displaying the number of migrated breast cancer cells treated with 5 mM L-lactate. **(I)** Western blot analysis indicating the total L-lactylated levels of lysine in MDA-MB-231 and CAL-51 cells treated with 5 mM L-lactate. **(J)** Western blot analysis for the total L-lactylated levels of lysine in MDA-MB-231 and CAL-51 cells after DTL knockdown. Data in **D, F, G** and **H** were presented as mean ± S.D (n = 3). Two-tailed Student’s *t*-test.

To reveal the mechanism how DTL governs malignant development of BC, and what role of reprogrammed glycolysis during the regulatory process, we performed GO and KEGG enrichment analysis on differentially expressed genes in BC patients with low and high DTL expression. As the volcano plot indicated, 609 up-regulated and 359 down-regulated genes were obtained (**Figure 6A**). Moreover, these changed genes were enriched to multiple biological processes (BP), molecular functions (MF) and cellular components (CC), mainly focusing on cell cycle-associated-regulatory processes (**Supplementary Figures S8A-C**). Consistently, depleted DTL expression resulted in decreased distribution ratio of breast cells in S phase and apparent arrest in G_2_/M phase, suggesting a vital function in the regulation of DTL on cell cycle progression (**Supplementary Figures S9A, B**). KEGG analysis illustrated various alternative pathways such as MYC, glycolysis and epithelial-mesenchymal transitions (**Figure 6B**). Among them, it’s notable that MYC is a crucial regulator of stemness, and we firstly investigated the influence of DTL on stemness-associated genes statuses. As expected, the expression of stemness-associated genes including *ABCG2*, *SOX9*, *NANOG* and *SOX2* was down-regulated observably (**Figures 6C, D**).

L-lactate is the end-product of glycolysis and participates in the regulation of various biological processes involving in metabolism, stemness and immune response etc. We subsequently investigated the regulatory effect of L-lactate on stem cells-properties and found that L-lactate could promote the expression of stemness-associated genes including *ABCG2*, *NANOG* and *SOX2* (**Figures 6E, F**). In addition, L-lactate was able to enhance proliferative capacity and migrated potential of BC cells (**Figures 6G, H**; **Supplementary Figures S10A, B**). Importantly, L-lactate was capable of rescuing partly proliferative and migrated ability of BC cells with DTL depletion (**Supplementary Figures S10C-F**).

The literature has reported that lactate acid could provide carbons to the acetyl-residues of proteins, leading to lactylated modification post-translationally of these proteins, which plays essential roles in the tumorigenesis and development of various cancers including breast cancer (24–26). Hence, we first treated MDA-MB-231 and CAL-51 cells with 5 mM lactate and as the Western blot data shown, the levels of total lactylated lysines were significantly boosted of these cells (**Figure 6I**). Interestingly, DTL knockdown was associated with a partial reduction in L-lactylated levels of lysines across BC cell models. Notably, this effect was most evident in the construction 3 of MDA-MB-231 cells (**Figure 6J**), suggesting that L-lactylation modification may serve as a potential regulator for BC progression driven by L-lactate.

## Discussion

CRLs demonstrate marked dysregulation across malignancies, positioning them as promising therapeutic targets in oncology (27). CRLs orchestrate multifaceted oncogenic programs through the regulation of energy metabolism, autophagic flux, EMT plasticity, stemness maintenance, angiogenic signaling, inflammatory cascades, senescence bypass, immune evasion, and therapy resistance (14, 28). In our pan-cancer analysis, we observed that the expression of the CRL family member DTL was upregulated in multiple tumor tissues, with a particularly pronounced increase in BC. Additionally, we found that the upregulation of DTL in BC tissue samples is not limited to the mRNA level. Both analyses of public cohorts and immunohistochemical staining indicated that the protein expression of DTL in BC tissues is significantly higher than in normal breast tissues, corroborating prior reports (29–31). Other studies have illuminated that DTL was involved in the tumorigenesis as well as in the proliferation, migration, and invasion of cancer cells (32–36). Also, high expression of DTL often predicts poor prognosis of varied tumors, such as hepatocellular carcinoma (37), gastric cancer (33), bladder cancer (35), melanoma (13), and nasopharyngeal carcinoma (38). Our study also revealed similar findings, with both bioinformatics analysis and clinical cohorts indicating that high expression of DTL is associated with poor prognosis in BC patients.

Mechanistic dissection of DTL dysregulation in BC identified two predominant drivers involving in gene amplification and promoter hypomethylation. Gene amplification, defined as an increase in the copy number of a specific chromosomal region, is a well-documented mechanism contributing to drug resistance and has been implicated in the clinical prognosis and diagnostic potential of various cancers (39). Breast cancer, in particular, is marked by substantial epigenetic reconfigurations (40). Hypermethylation is predominantly observed in CpG islands and their adjacent shores, correlating with the silencing of tumor suppressor genes, while hypomethylation is prevalent in intragenic regions, potentially leading to the activation of oncogenes (40, 41). While our study design precludes causal inference, the observed DTL promoter hypomethylation in tumors aligns with the well-established paradigm wherein promoter hypomethylation facilitates transcriptional activation (42–44). Our analysis of BC samples has identified a high incidence of gene amplification events and decreased promoter methylation at the DTL locus (**Figures 2A, F**), which may be instrumental in the dysregulation of DTL expression. Furthermore, the impact of tumor mutational burden (TMB) and mutant allele tumor heterogeneity (MATH) on the phenotypic presentation of BC warrants consideration. Elevated TMB, reflecting a higher number of somatic mutations within a tumor, has been associated with increased neoantigen production and a more aggressive disease phenotype (45). Similarly, high levels of MATH, indicating a greater diversity of mutant alleles within the tumor, can influence tumor evolution and response to therapy (46). Ma D et al. (2017) demonstrated that in hormone receptor-positive, HER2-negative BC patients, higher MATH scores were associated with a tendency toward worse overall survival and an increased frequency of TP53 mutations (47). And our study discovered that in BC samples, the MATH scores were significantly higher in the high DTL expression group compared to the low DTL expression group (**Figure 2E**), suggesting that DTL expression might be a significant factor in the generation of genetic heterogeneity within BC and could potentially influence patient prognosis and response to treatment.

Mechanistically, our experiments confirms that DTL promotes the progression of BC by accelerating the production of L-lactate. L-lactate, an end product of glycolysis, has emerged as a critical metabolite that actively participates in tumorigenesis beyond its traditional role as an energy substrate (48). Its accumulation within the tumor microenvironment has been implicated in several mechanisms that collectively drive cancer progression. Firstly, L-lactate modulates the tumor extracellular pH by creating an acidic niche, which can shield cancer cells from immune surveillance and enhance their invasive capabilities (49). This acidic shift can also activate various oncogenes and repress tumor suppressor functions, thereby promoting a more aggressive cancer phenotype (50). Secondly, L-lactate serves as an energy source for oxidative phosphorylation in cancer cells, particularly in regions of the tumor where oxygen is scarce, thus supporting the metabolic plasticity necessary for tumor survival and growth under hypoxic conditions (51). Thirdly, there is evidence suggesting that L-lactate can directly influence the activity of certain signaling pathways such as the mTOR pathway, which regulates cell growth and proliferation (52). By activating these pathways, L-lactate may enhance tumor cell proliferation and metabolism. Moreover, L-lactate has been shown to promote endothelial-cell activation and angiogenesis via both HIF-dependent and HIF-independent pathways (53). This pro-angiogenic effect ensures the formation of new blood vessels, which are essential for nourishing the growing tumor and facilitating the escape of cancer cells into the circulation, thus contributing to metastasis (54). Lastly, L-lactate can influence the function of immune cells within the tumor microenvironment. It has been reported to induce a phenotype in immune cells that favors immunosuppression, potentially by activating regulatory T cells (Tregs) and suppressing the activity of cytotoxic T lymphocytes, thereby dampening the antitumor immune response (55, 56). Additionally, both the research by Wang C et al. and Mu X et al. have demonstrated that tumor-derived L-lactate can regulate the polarization of inflammatory macrophages in BC, thereby promoting tumor progression (57, 58). In summary, consistent with our experimental findings (**Figures 6D–J**), high expression of DTL correlates with increased lactate levels in the tumor microenvironment, leading to accelerated tumor growth and proliferation. Our findings elucidate the multifaceted role of DTL in BC progression through its regulation of L-lactate production, which in turn coordinates a complex interplay of biological processes that collectively drive tumor aggressiveness. These insights underscore the potential of targeting DTL and L-lactate metabolism as a therapeutic strategy in BC.

As an E3 ubiquitin ligase, DTL may regulate L-lactate biosynthesis through two distinct ubiquitination modalities, canonical degradative pathways and non-proteolytic mechanisms. As for the canonical ubiquitin-mediated degradation pathway, the research by Su Y et al. offers a valuable perspective, demonstrating that the assembly of the CRL4^COP1^ E3 ligase induced by glucose can lead to the ubiquitination and subsequent degradation of p53, leading to the derepression of glycolytic enzymes and ultimately resulting in increased L-lactate production (59). While no significant correlation was observed between DTL expression and TP53 expression in BC samples in our study, we did note that the prevalence of TP53 mutations was markedly higher in the group with high DTL expression than in the group with low DTL expression (**Figure 3C**). Moreover, both previous research and the results of our study indicated that in BC samples with TP53 mutations, the expression levels of LDHA, ENO1, and PKM2 were significantly higher than in BC samples with wild-type TP53 (60, 61). These findings highlight the need to investigate DTL’s role in TP53-mutant BC pathobiology, particularly its potential as a synthetic lethal target.

In the meantime, the non-degradative functions of CRLs have garnered increasing attention in recent years (8). As our experimental results indicate, DTL engages in direct interactions with specific glycolytic enzymes (**Figures 5I, J**; **Supplementary Figure S7B**). Given the positive correlation between DTL expression and the expression of most glycolytic enzymes in BC samples (**Figure 3D**; **Supplementary Figure S6**), this suggests that DTL is not functioning through ubiquitin-mediated degradation in this context. The diversity of ubiquitin chain types is crucial for their distinct cellular functions. Although K48-linked chains generally target proteins for degradation (62, 63), other linkages like K63 and M1 are known for non-degradative roles in processes such as immune signaling and protein homeostasis (64, 65). Emerging evidence also points to specific functions for less characterized linkages, such as K6 in mitophagy and K27 in innate immunity, indicating a complex regulatory network that extends beyond proteolysis (66, 67). Additionally, the traditional view that lysine is the primary target for protein ubiquitylation has faced significant challenges recently. Instead, alternative sites such as cysteine (C), serine (S), and threonine (T) have been identified as functional ubiquitylation targets, forming thioester and hydroxyester linkages (68). Recent studies have also increasingly focused on the non-degradative functions of CRLs. For instance, the CRL3^SPOP^ complex has been shown to regulate genomic stability by non-degradative ubiquitinating Geminin during the cell cycle, thereby ensuring that DNA replication occurs only once per cell cycle (69, 70). In the context of cancer, the non-degradative functions of CRLs are particularly relevant. The CRL3^SPOP^ complex has been shown to suppress tumorigenesis by non-degradative ubiquitinating several oncoproteins, including HIPK2 and INF2 (71, 72). This modification enhances HIPK2’s ability to phosphorylate HP1γ, promoting DNA repair, and inhibits INF2-mediated mitochondrial fission, thereby reducing the proliferation and invasion of cancer cells. The emerging appreciation for these non-degradative roles of CRLs underscores the complexity of their contributions to the pathobiology of diseases, including cancer, and highlights the need for further research to fully elucidate their mechanisms of action and potential as therapeutic targets. Regrettably, no studies on the non-degradative functions of DTL have been identified in the current literature search. Although the PLA data demonstrated that DTL could directly interact with glycolytic enzymes such as ENO1 and LDHA, with particularly strong binding potential observed for LDHA, the hypothesis proposing a non-proteolytic role of DTL in metabolic regulation requires further experimental validation. Additional investigations, including co-immunoprecipitation assays to confirm potential post-translational modifications mediated by these interactions, would be essential to substantiate this mechanistic hypothesis. The discovery of LDHA Y10 phosphorylation as a metastatic driver (Jin, L et al., 2017) opens new avenues for investigating DTL’s non-canonical roles (73). Future studies employing phospho-specific LDHA mutants and ubiquitination-editing tools will clarify whether DTL sustains Y10 phosphorylation via kinase stabilization or phosphatase degradation.

Our study advances understanding of DTL in BC pathogenesis, yet several limitations warrant consideration. While our cohort size was statistically robust for initial hypothesis testing, its moderate sample size and restricted demographic diversity—particularly in terms of ethnicity, tumor subtypes, and clinical stages—may constrain the broader applicability of our findings. The reliance on public repositories (TCGA and GTEx), though advantageous for data accessibility, introduces inherent batch effects and technical variability across sequencing platforms that could influence biomarker validation. Methodologically, the cross-sectional design and retrospective data collection preclude definitive conclusions regarding causal relationships or longitudinal progression patterns in DTL-associated oncogenesis. Mechanistically, while our bioinformatic models and pathway analyses suggest potential interactions between DTL and key glycolytic regulators (e.g., ENO1, LDHA), the precise molecular binding interfaces and post-translational modifications require orthogonal validation through structural biology approaches like cryo-EM or X-ray crystallography. Furthermore, our integrated genetic-epigenetic framework, though comprehensive, may not fully account for microenvironmental influences or non-coding regulatory elements that modulate DTL functionality *in vivo*. To address these gaps, prospective multicenter studies employing standardized multi-omics protocols in ethnically balanced BC cohorts are essential. Complementary functional studies using organoid models and CRISPR screening could elucidate DTL’s context-dependent roles while identifying druggable pockets for therapeutic development. While our findings establish DTL’s role through bioinformatics analyses and *in vitro* experiments, future studies will utilize animal models combined with advanced imaging to validate its effects on BC growth and metastasis in physiological contexts. These experiments aim to confirm whether the identified mechanisms are conserved *in vivo*, further bridging our findings to clinical relevance.

## Conclusion

DTL reprograms glycolysis by interacting with LDHA directly in breast cancer (BC) cells, leading to increased production of the oncometabolite L-lactate, which drives tumor progression (**Supplementary Figure S11**). Elevated DTL expression correlates with poor prognosis, tumor aggressiveness, and metabolic alterations that enhance cancer cell proliferation and migration, highlighting its key role in promoting BC development.

## Data Availability

The datasets presented in this study can be found in online repositories. The mRNA expression and subsequent analysis was in whole or part based upon data generated by the Cancer Genome Atlas Program (TCGA) research network (https://www.cancer.gov/tcga) and the Genotype-Tissue Expression (GTEx) Project (https://www.gtexportal.org). The proteomic expression profile was based upon data generated by the Clinical Proteomic Tumor Analysis Consortium (CPTAC) (https://proteomics.cancer.gov/programs/cptac).
