# Corrigendum: Rewired glycolysis by DTL accelerates oncometabolite L-lactate generation to promote breast cancer progression

**DOI:** 10.3389/fonc.2025.1630154

**Published:** 2025-06-27

**Authors:** Yuhao Liu, Jinting Li, Yiren Cao, Mengzhu Lv

**Affiliations:** ^1^ Key Laboratory of Carcinogenesis and Translational Research (Ministry of Education), Department of Radiation Oncology, Peking University Cancer Hospital and Institute, Beijing, China; ^2^ Key Laboratory of Carcinogenesis and Translational Research (Ministry of Education), Laboratory of Molecular Oncology, Peking University Cancer Hospital and Institute, Beijing, China

**Keywords:** DTL, glycolysis, L-lactate, breast cancer, progression

In the published article, there was an error in **Figure 6C** as published. We regrettably discovered an inadvertent duplication of loading control bands (β-actin and GAPDH for CAL-51 cell line) between **Figures 5A** and **Figure 6C** during figure reorganization. We wish to emphasize that the duplicated controls originated from concurrent experiments — both **Figures 5A** and **6C** datasets were generated during the same experimental batch, though logically separated into two figures to enhance manuscript readability. The corrected **Figure 6C** and its caption “Western blot analysis for the expression of stemness-associated genes in MDA-MB-231 and CAL-51 cells after DTL knockdown” appear below.

**Figure 6:**
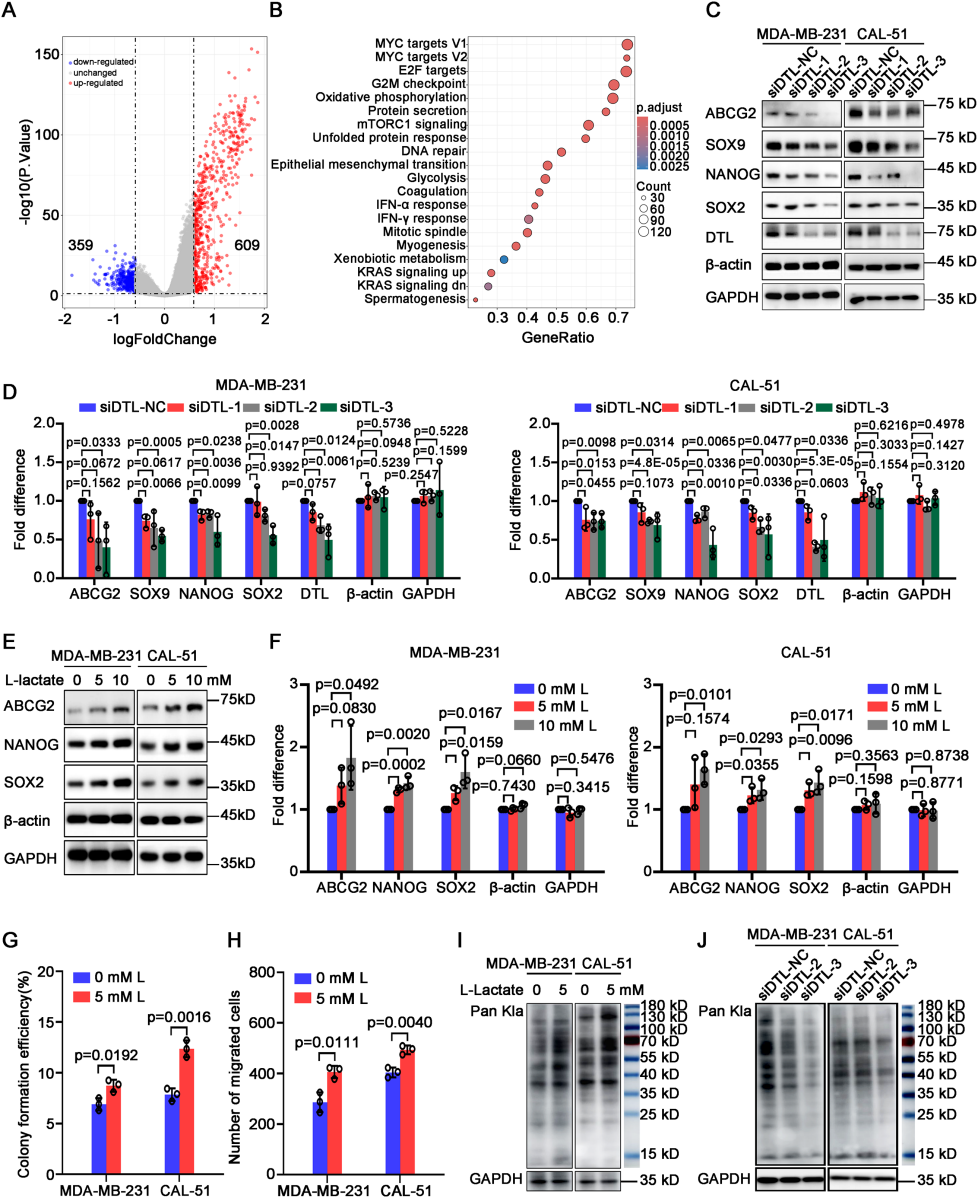
L-lactate supports cell proliferation and migration of breast cancer depending on DTL. **(A)** Volcano plot showing differentially expressed genes in breast cancer patients with low and high DTL expression. **(B)** Dot plot indicating the alternative signal pathways of KEGG enrichment analysis. **(C)** Western blot analysis for the expression of stemness-associated genes in MDA-MB-231 and CAL-51 cells after DTL knockdown. **(D)** Histogram showing the expression of indicated molecules after DTL knockdown in MDA-MB-231 and CAL-51 cells. **(E)** Immunoblotting analysis for the expression of stemness-related indicators in MDA-MB-231 and CAL-51 cells treated with different concentrations of L-lactate. **(F)** Histogram showing the levels of indicated molecules in MDA-MB-231 and CAL-51 cells treated with 5 mM L-lactate. **(G)** Histogram indicating the colony-forming efficiency of MDA-MB-231 and CAL-51 cells with 5 mM L-lactate treatment. **(H)** Histogram displaying the number of migrated breast cancer cells treated with 5 mM L-lactate. **(I)** Western blot analysis indicating the total L-lactylated levels of lysine in MDA-MB-231 and CAL-51 cells treated with 5 mM L-lactate. **(J)** Western blot analysis for the total L-lactylated levels of lysine in MDA-MB-231 and CAL-51 cells after DTL knockdown. Data in **D, F, G** and **H** were presented as mean ± S.D (n = 3). Two-tailed Student’s *t*-test.

The authors apologize for this error and state that this does not change the scientific conclusions of the article in any way. The original article has been updated.

