# Supplementary material for: Rewired glycolysis by DTL accelerates oncometabolite L-lactate generation to promote breast cancer progression
Source: Front Oncol. 2025 May 5;15:1583752. doi: 10.3389/fonc.2025.1583752 (PMC12086152; doi:10.3389/fonc.2025.1583752)
Supplement: Supplementary file 1 [file DataSheet1.docx]

**Supplementary figures**

**Supplementary figure 1**


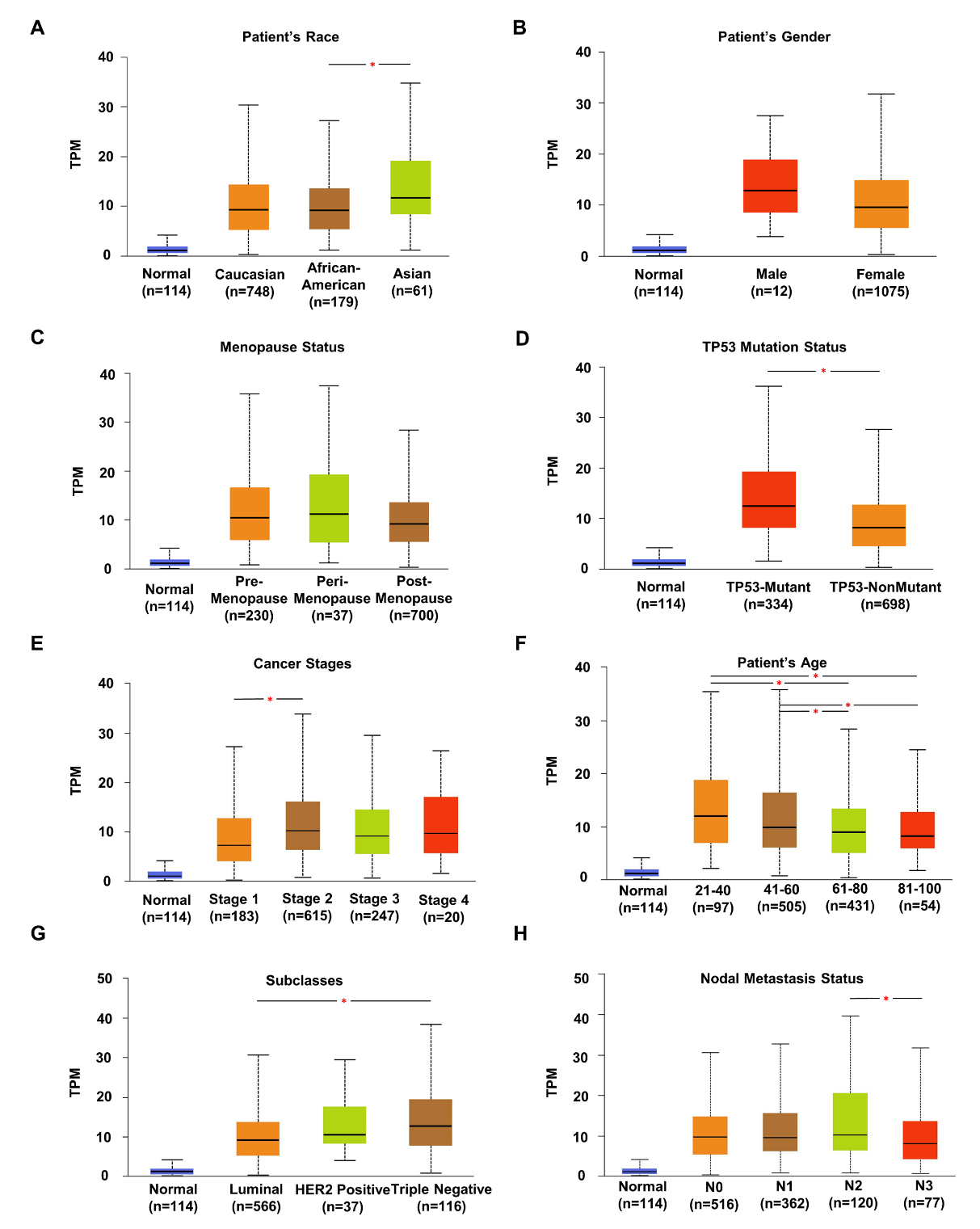


**Supplementary figure 1. DTL expression in patients of breast cancer stratified according to different clinicopathological features.**

Breast cancer patients were stratified by **(A)** race, **(B)** gender, **(C)** menopause status, **(D)** TP53 mutation status, **(E)** cancer stage, **(F)** age, **(G)** subclasses, and **(H)** nodal metastasis status. In this figure, there was a significant difference between DTL expression in normal group and any patient group, so the difference was not specifically labelled. If there was a significant difference (p<0.05) between any two patient groups, the difference would be marked with *.

**Supplementary figure 2**


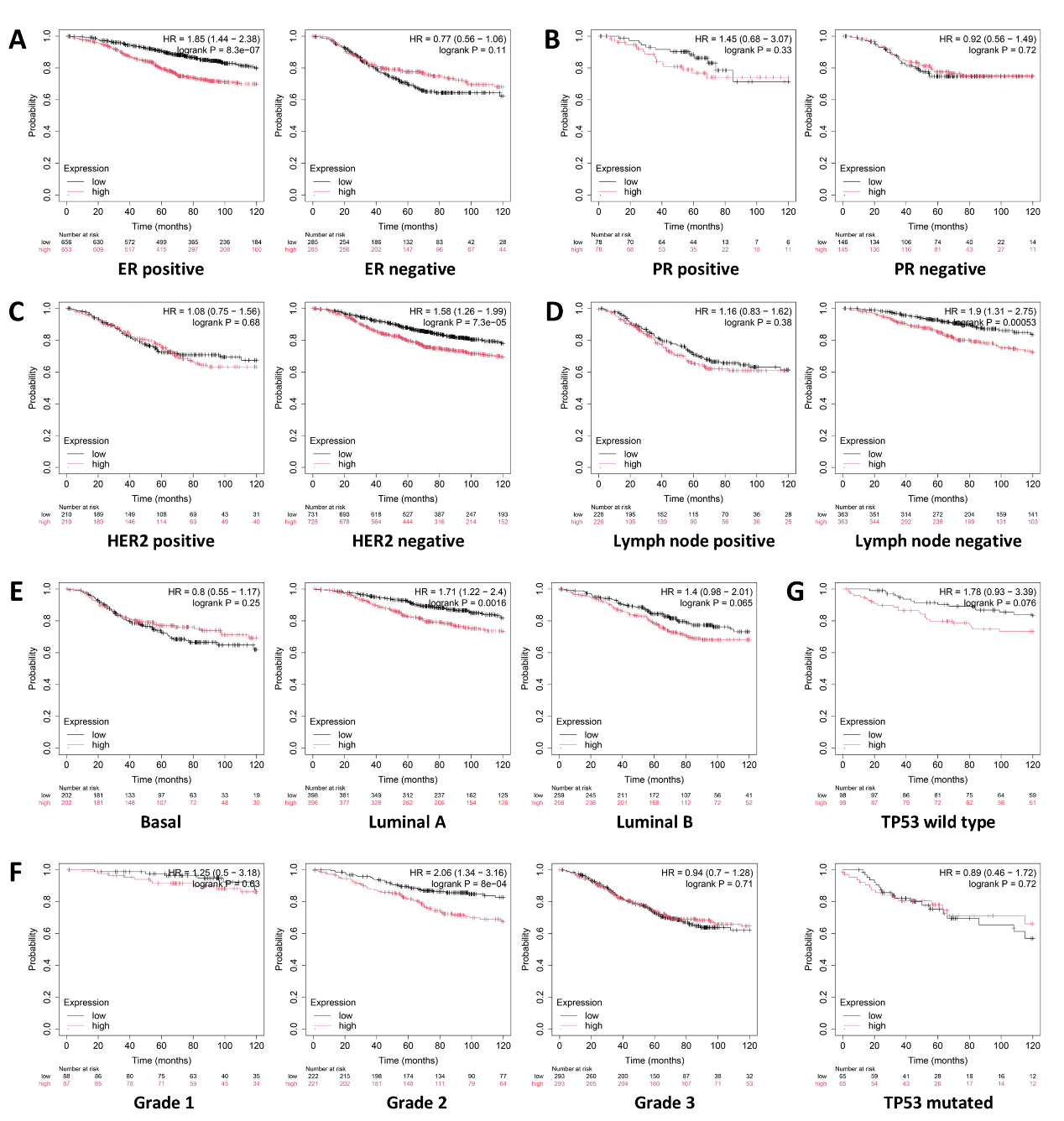


**Supplementary figure 2. The impact of high and low DTL expression on overall survival (OS) of breast cancer patients with different clinical characteristics.**

Breast cancer patients were stratified by **(A)** ER status, **(B)** PR status, **(C)** HER2 status, **(D)** lymph node status, **(E)** subtype, **(F)** grade, and **(G)** TP53 status. In the above analyses, patients were stratified into high and low DTL expression groups based on the median DTL expression level.

**Supplementary figure 3**


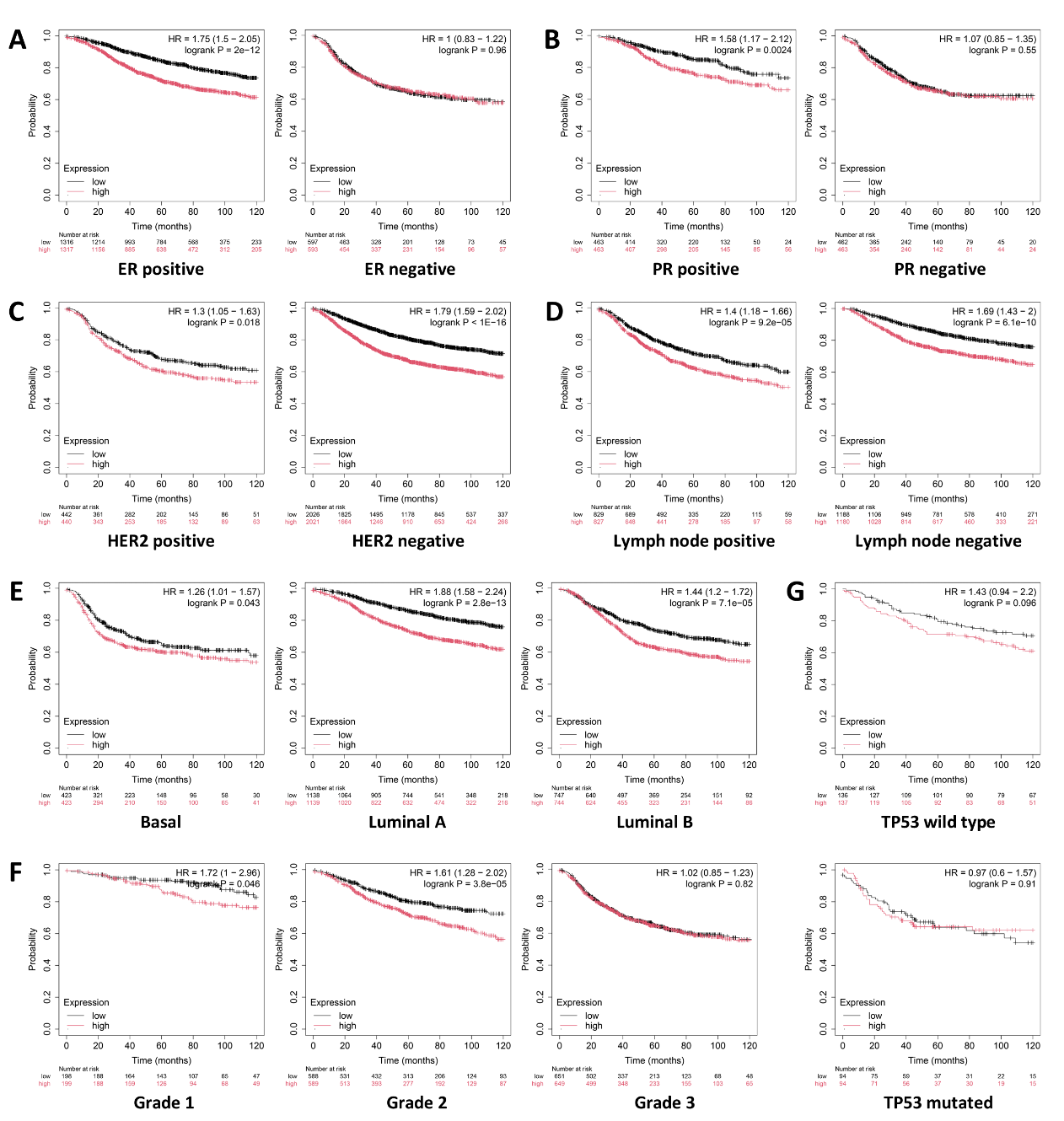


**Supplementary figure 3. The impact of high and low DTL expression on relapse-free survival (RFS) of breast cancer patients with different clinical characteristics.**

Breast cancer patients were stratified by **(A)** ER status, **(B)** PR status, **(C)** HER2 status, **(D)** lymph node status, **(E)** subtype, **(F)** grade, and **(G)** TP53 status. In the above analyses, patients were stratified into high and low DTL expression groups based on the median DTL expression level.

**Supplementary figure 4**


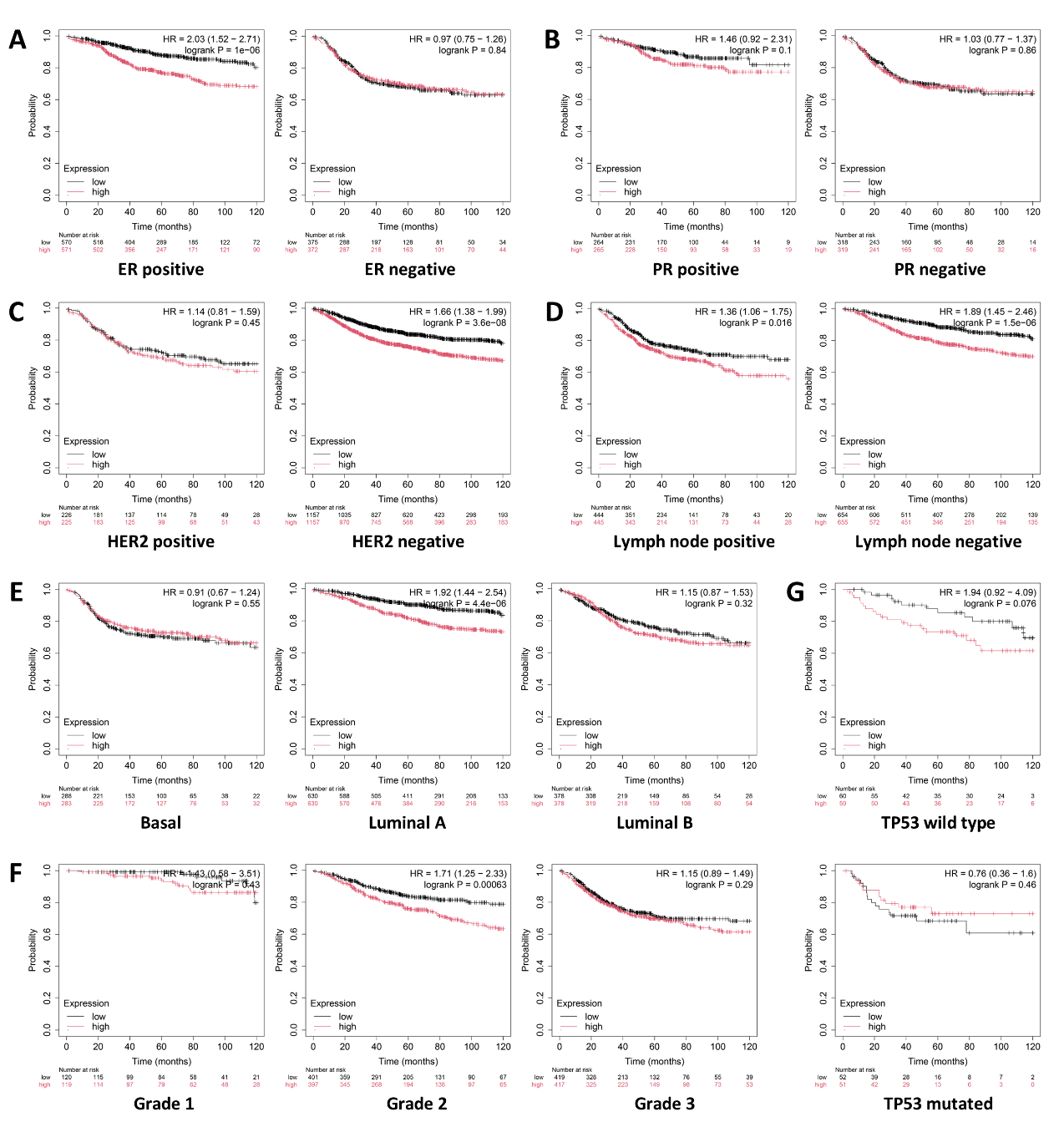


**Supplementary figure 4. The impact of high and low DTL expression on metastasis-free survival (DMFS) of breast cancer patients with different clinical characteristics.**

Breast cancer patients were stratified by **(A)** ER status, **(B)** PR status, **(C)** HER2 status, **(D)** lymph node status, **(E)** subtype, **(F)** grade, and **(G)** TP53 status. In the above analyses, patients were stratified into high and low DTL expression groups based on the median DTL expression level.

**Supplementary figure 5**


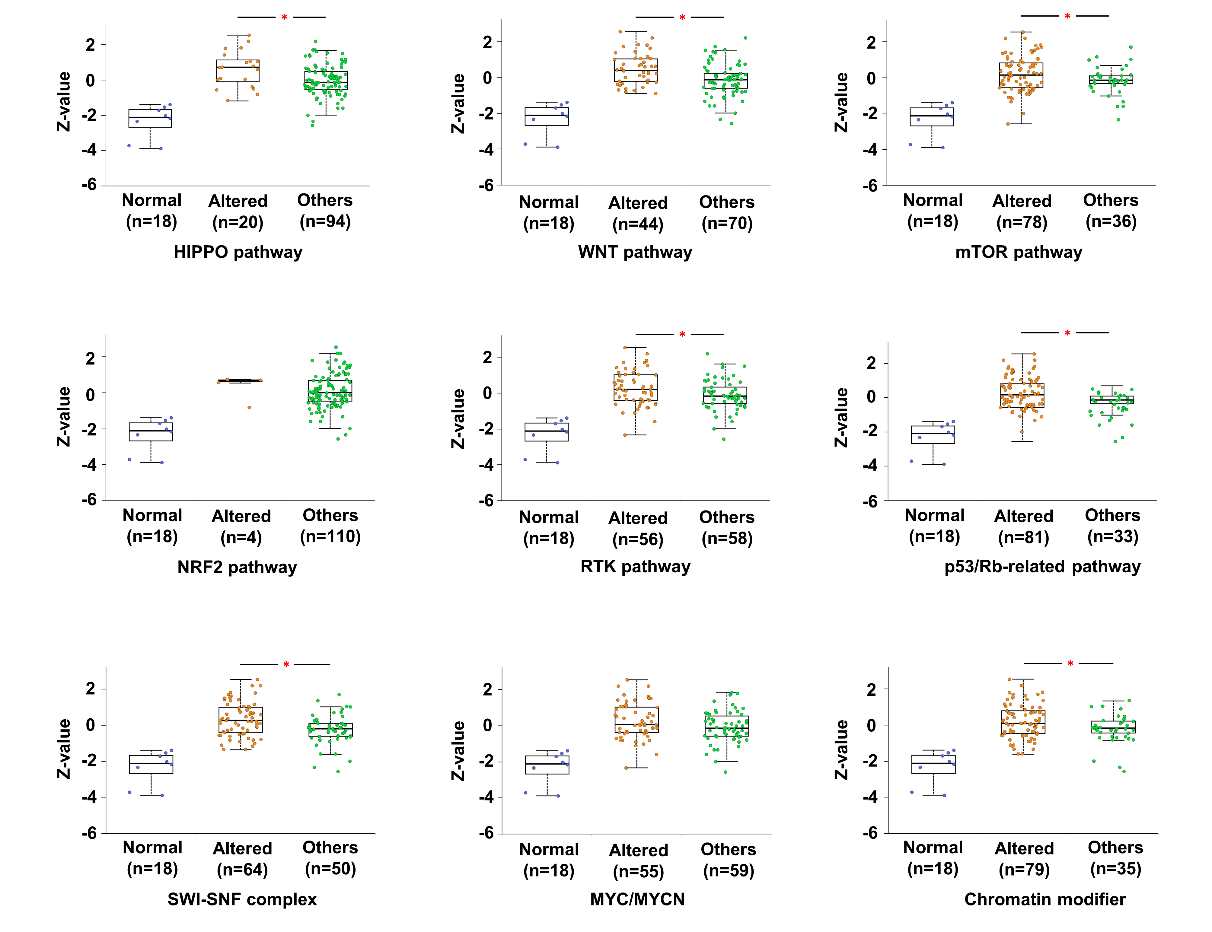


**Supplementary figure 5. Association of DTL expression with alterations in pivotal cancer-related regulatory pathways.**

In this figure, there was a significant difference between DTL expression in normal group and any patient group, so the difference was not specifically labelled. If there was a significant difference (p<0.05) between altered group and other group, the difference would be marked with *.

**Supplementary figure 6**


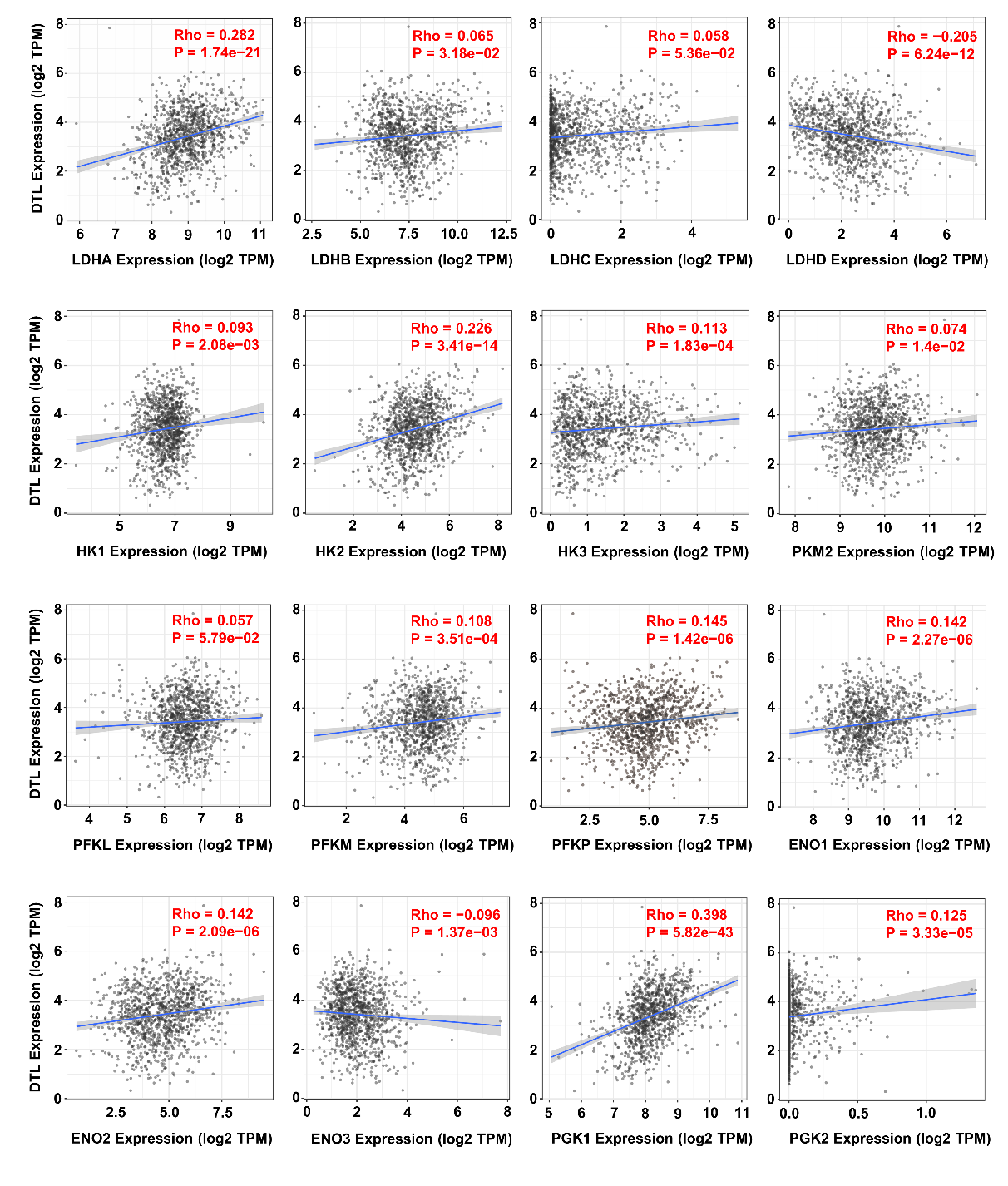


**Supplementary figure 6. Expressing correlation of DTL with key enzyme genes during glycolysis in tumor cells.**

The correlation of DTL with key enzyme genes in tumor metabolism showing the Spearman's Rho value and statistical significance. Log2 TPM is the log2 of the Transcript Count Per Million.

**Supplementary figure 7**

**
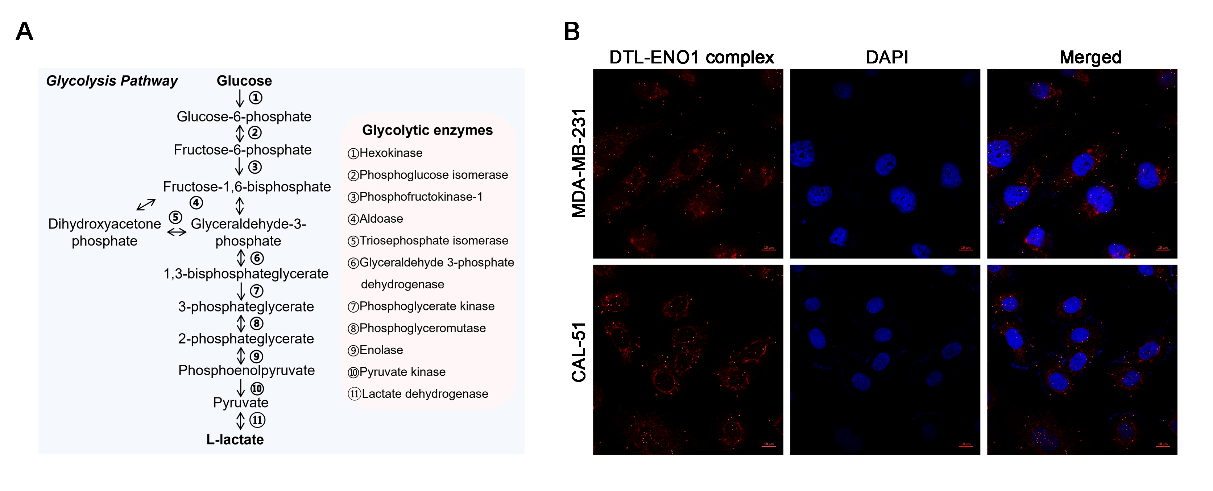
**

**Supplementary figure 7. DTL takes responsibility for rewired glycolysis of breast cancer cells.**

**(A)** Schematic diagram of glycolysis pathway and glycolytic enzymes. **(B)** Proximity ligation assay (PLA) showing the direct interaction between DTL and ENO1 in breast cancer cells of MDA-MB-231 and CAL-51. Nuclei were stained with DAPI. Scale bar, 10 μm.

**Supplementary figure 8**


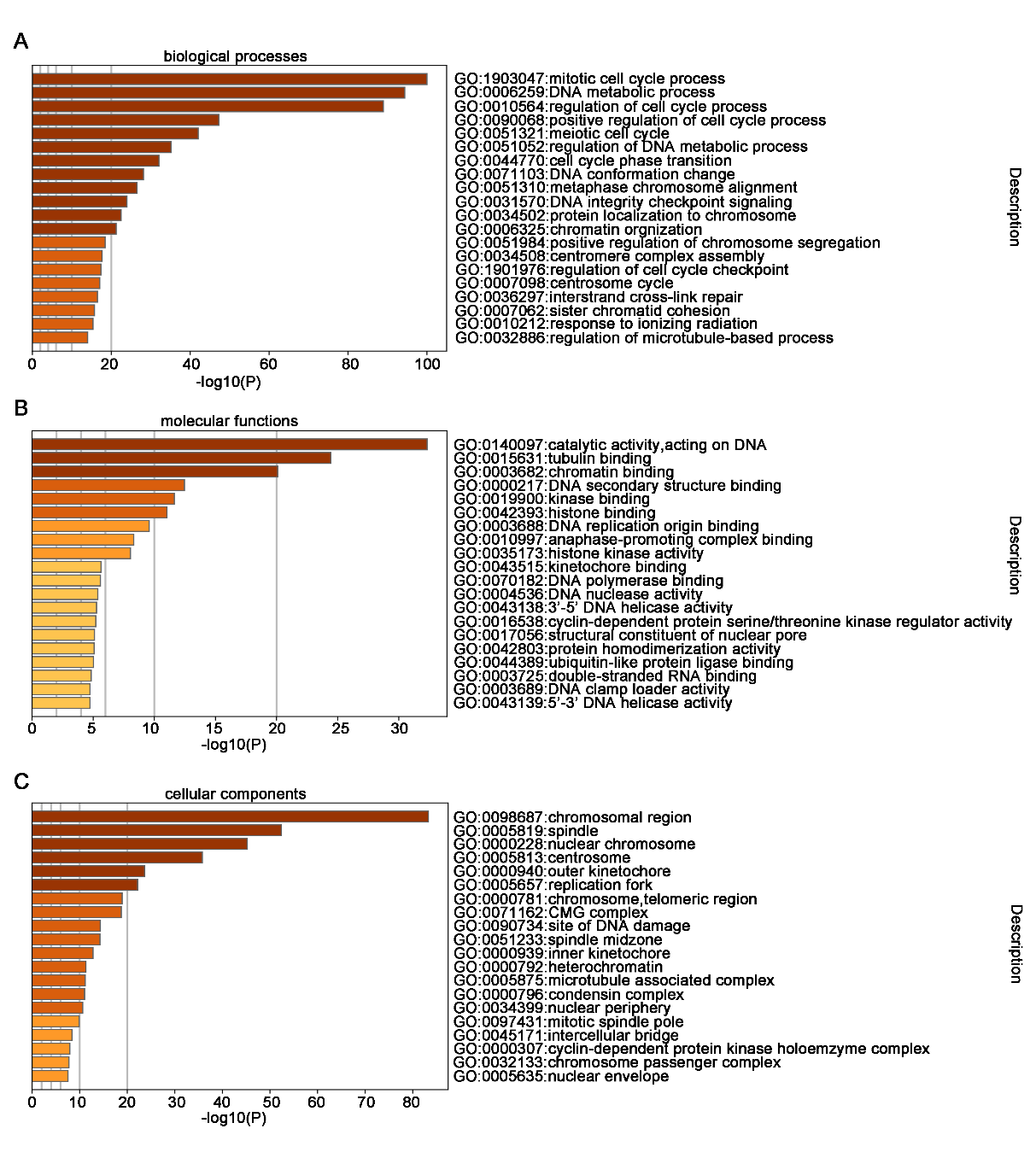


**Supplementary figure 8. DTL plays a crucial role in the regulation of cell cycle.**

Differentially enriched pathways by GO analysis involved in biological processes **(**A), molecular functions **(B)** and cellular components **(C)**.

**Supplementary figure 9**


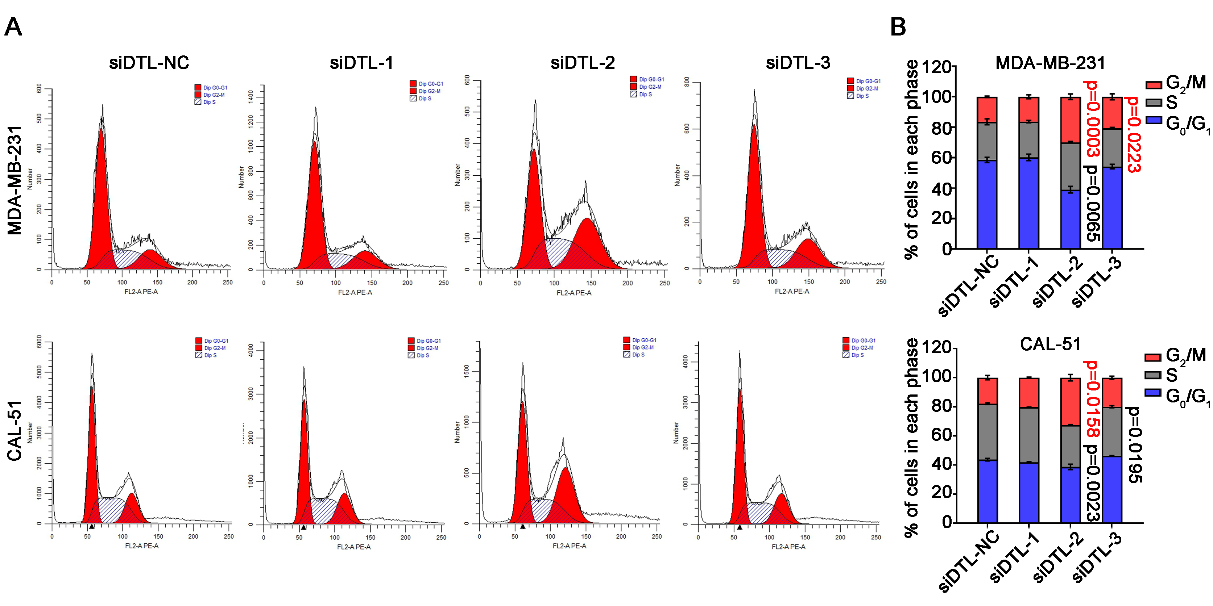


**Supplementary figure 9. DTL plays an important role in the regulation of cell cycle progression of breast cancer cells.**

**(A)** Cell cycle analysis of MDA-MB-231 and CAL-51 cells after DTL knockdown using PI staining. **(B)** Histograms showing the ratio of MDA-MB-231 and CAL-51 cells distributed in each phase of cell cycle. Data were presented as mean ± SD. Unpaired two-tailed Student’s *t*-test was used for statistical analysis.

**Supplementary figure 10**

**
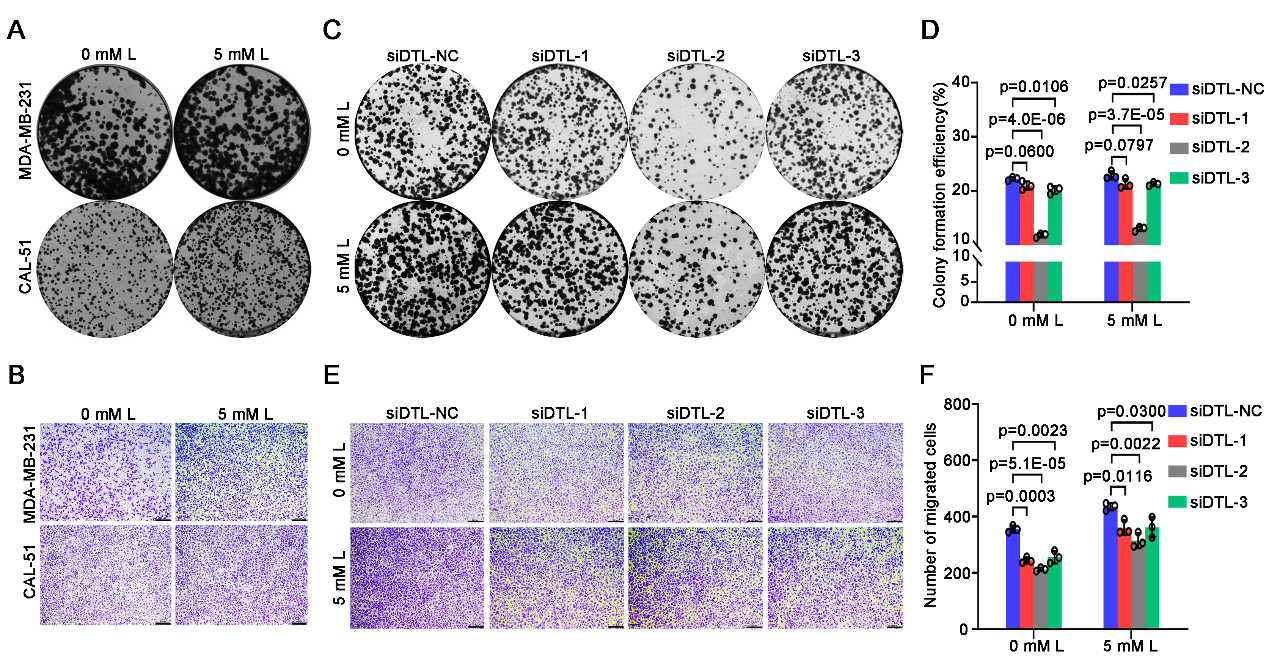
**

**Supplementary figure 10. L-lactate supports cell proliferation and migration of breast cancer depending on DTL.**

**(A)** Representative micrographs showing the grown colonies formed by MDA-MB-231 and CAL-51 cells treated with 5 mM L-lactate. **(B)** Representative photographs of migrated MDA-MB-231 and CAL-51 cells with L-lactate treatment. Scale bar, 100 μm. **(C)** Photographs of the colonies formed by DTL-depleted CAL-51 cells treated with 5 mM L-lactate. **(D)** Histogram exhibiting the changed colony-forming efficiencies of CAL-51 cells with DTL knockdown after 5 mM L-lactate treatment. **(E)** Transwell assay to measure the changes of migrated potential in DTL-depleted CAL-51 cells treating with 5 mM L-lactate for 48 hours. Scale bar, 100 μm. **(F)** Histogram demonstrating the number of migrated CAL-51 cells with DTL knockdown after L-lactate treatment. Data in **D** and **F** were the mean ± SD. Unpaired two-tailed Student’s *t*-test was used for statistical analysis.

**Supplementary figure 11**

**
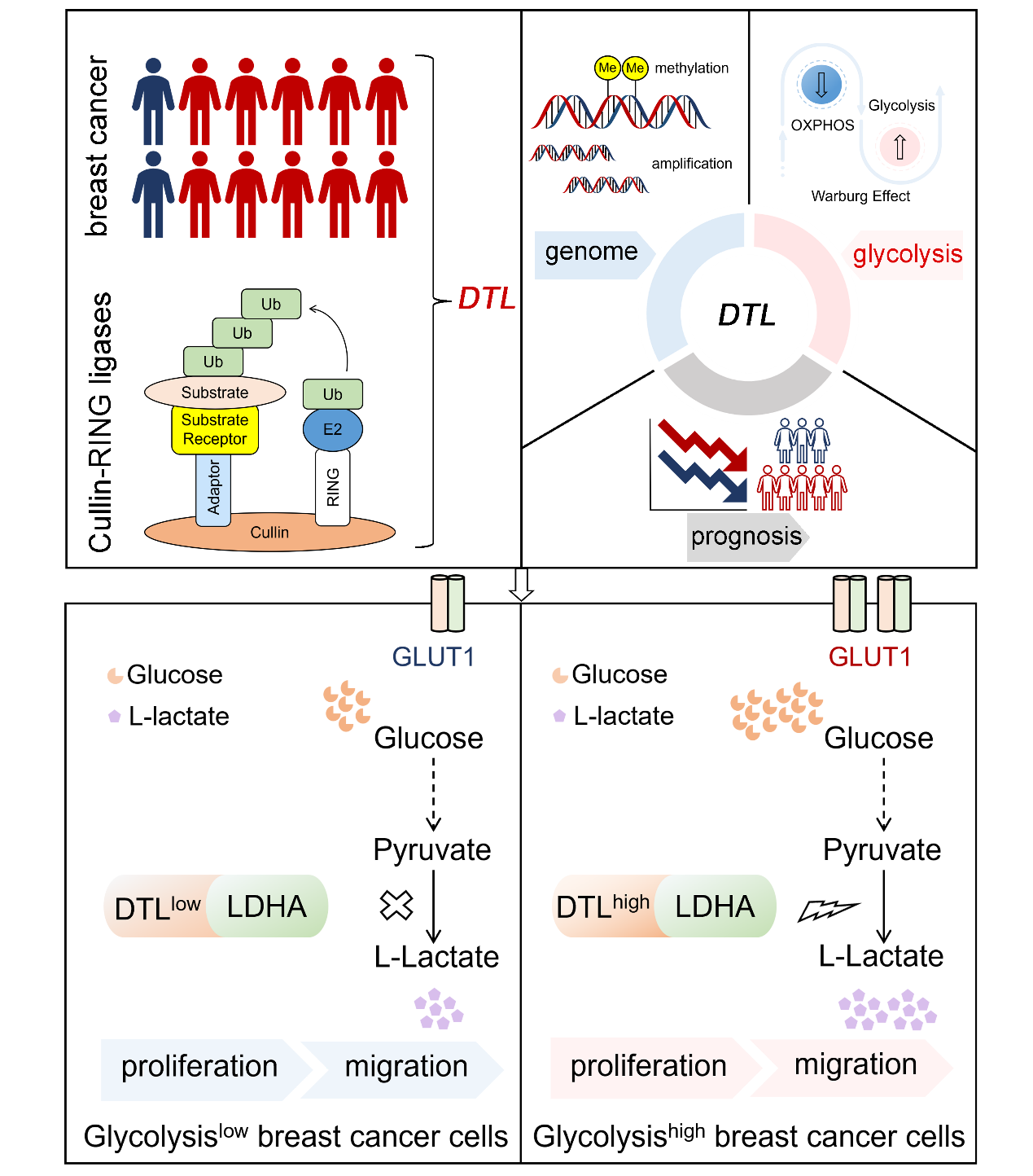
**

**Supplementary figure 11. Schematic diagram of the mechanism that DTL rewires glycolysis to generate L-lactate for breast cancer progression.**

**Supplementary tables**

**Supplementary table 1. siRNA sequences used in this study.**

| siRNA | target sequence (5’ to 3’) |
| --- | --- |
| DTL-1 | GAAGCTGCCTACATATGGA |
| DTL-2 | GTATGGGATTTACGTAAGA |
| DTL-3 | GGACTTCTGTGGTCCTGAA |

**Supplementary table 2. Primary antibodies of Western Blot used in this study.**

| Name | Vendor | Catalog No. | WB |
| --- | --- | --- | --- |
| GLUT1 | Proteintech | 21829-1-AP | 1:1000 |
| HK2 | Proteintech | 22029-1-AP | 1:1000 |
| ENO1 | ABclonal | A1033 | 1:1000 |
| PKM2 | Proteintech | 15822-1-AP | 1:1000 |
| LDHA | Proteintech | 19987-1-AP | 1:1000 |
| ABCG2 | Abcam | Ab108312 | 1:2000 |
| SOX9 | CST | 82630T | 1:1000 |
| NANOG | CST | 4903S | 1:1000 |
| SOX2 | CST | 3579S | 1:1000 |
| L-Lactyl Lysine | PTM BIO | PTM-1401RM | 1:1000 |
| DTL | Abcam | ab72264 | 1:1000 |
| GAPDH | Proteintech | 60004-1-Ig | 1:10000 |
| β-actin | Abcam | Ab8226 | 1:5000 |
